# Global patterns of insect diversification: towards a reconciliation of fossil and molecular evidence?

**DOI:** 10.1038/srep19208

**Published:** 2016-01-18

**Authors:** Fabien L. Condamine, Matthew E. Clapham, Gael J. Kergoat

**Affiliations:** 1 CNRS, UMR 5554 Institut des Sciences de l’Evolution (Université de Montpellier), Place Eugène Bataillon, 34095 Montpellier, France; 2 University of Alberta, Department of Biological Sciences, Edmonton T6G 2E9, AB, Canada; 3 Department of Earth and Planetary Sciences, University of California, Santa Cruz, CA 95064, USA; 4 INRA, UMR 1062 CBGP (INRA, IRD, CIRAD, Montpellier SupAgro), 755 Avenue du Campus Agropolis, 34988 Montferrier-sur-Lez, France

## Abstract

Macroevolutionary studies of insects at diverse taxonomic scales often reveal dynamic evolutionary patterns, with multiple inferred diversification rate shifts. Responses to major past environmental changes, such as the Cretaceous Terrestrial Revolution, or the development of major key innovations, such as wings or complete metamorphosis are usually invoked as potential evolutionary triggers. However this view is partially contradicted by studies on the family-level fossil record showing that insect diversification was relatively constant through time. In an attempt to reconcile both views, we investigate large-scale insect diversification dynamics at family level using two distinct types of diversification analyses on a molecular timetree representing *ca.* 82% of the extant families, and reassess the insect fossil diversity using up-to-date records. Analyses focusing on the fossil record recovered an early burst of diversification, declining to low and steady rates through time, interrupted by extinction events. Phylogenetic analyses showed that major shifts of diversification rates only occurred in the four richest holometabolous orders. Both suggest that neither the development of flight or complete metamorphosis nor the Cretaceous Terrestrial Revolution environmental changes induced immediate changes in diversification regimes; instead clade-specific innovations likely promoted the diversification of major insect orders.

“*Insects are not an invisible part of the fossil record. They have a rich history that must be reconciled with evolutionary interpretations of the taxonomic diversity and morphological and ecological disparity of modern insects.”* Labandeira and Sepkoski^1^

With at least one million species worldwide^2^, insects represent more than half of all known living organisms, which make them one of the major ecological and evolutionary radiations on Earth. They evolved into a hyperdiverse fauna that currently occupies almost every ecological niche, thanks to great diversity of life forms and developmental strategies^3^. The diversity of extinct insect species is well documented with a fossil record extending back more than ~410 million years ago (Myr ago)^1^,^3^,^4^. During that time they have survived the most severe mass extinctions and evolved against the backdrop of radical alterations in terrestrial floras, continental rearrangements, and changes in key environmental parameters, such as average global temperatures (Fig. 1). Throughout their evolution, insects have also developed a suite of morphological characteristics from complex mouthparts, to wings, and complete metamorphosis that probably allowed them to adapt in a continuously changing world.

Multiple hypotheses have been proposed to account for insect diversity dynamics^5^. The most common hypotheses propose that insect diversity either results from: (*i*) low extinction rates and resilience to mass extinctions; (*ii*) the acquisition of key innovations that allowed them to radiate into newly formed niches, or (*iii*) the appearance of new niches that favoured the diversification of some clades^1^,^3^,^5^,^6^. However, evidence is generally conflicting or equivocal, especially when comparing distinct sources such as time-calibrated molecular phylogenies or fossil records^5^. Phylogenies and the fossil record provide two complementary windows for understanding the temporal variations in biodiversity. Nonetheless most molecular studies generally focus on specific groups or on particular timeframes^6^,^7^,^8^,^9^,^10^,^11^,^12^,^13^,^14^, which precludes investigating and understanding what is behind the tremendous insects’ biodiversity? Furthermore, only mixed support has been provided for the effect of mass extinctions; for instance most fossil-based studies recovered an effect for the Permian-Triassic (P-T) mass extinction^1^,^4^, while phylogenetic studies suggested that it had no impact^14^. The role of other comparable events, such as the Triassic-Jurassic (T-J) mass extinction or the Cretaceous-Palaeogene (K-Pg) mass extinction, is less obvious or not detectable at the family-level or above^15^. Similarly, the impact of the environmental changes associated with the angiosperm radiation during the Cretaceous Terrestrial Revolution (KTR) *ca.* 125-85 Myr ago^16^,^17^ cannot be fully characterized because studies on distinct groups have yielded very contrasting patterns. Molecular phylogenetic studies focusing on specific groups indicate that groups like ants^7^, assassin bugs^18^, bees^19^, butterflies and moths^11^, orthopterans^20^, and various beetle groups (such as scarabs^12^ or weevils^9^, or as a whole^8^,^14^) appear to have diversified extensively during the KTR in response to newly formed niches^5^,^6^. However, other groups such as fleas^21^, flies^10^ and other beetle lineages (darkling beetles^13^ and stag beetles^22^) were not detectably more successful during this interval. Past global assessments of the fossil record also provided mixed evidence for a major diversification period associated with the KTR^1^. Only a few studies, such as a study on leaf-mining lepidopterans^23^, supported the role of angiosperm radiation, while others did not manage to find convincing evidence^4^,^24^,^25^.

Of particular interest are the results of two recent studies that postulated that the development of wings (Pterygota) and complete metamorphosis (Holometabola) have strongly influenced insect diversity dynamics. In the first study^26^, the authors relied on a family-level time-calibrated phylogeny including 82% of the insect families and used a maximum likelihood method (MEDUSA) that is explicitly designed to identify upshifts and downshifts of diversification rates on a dated phylogeny^27^. They identified 45 shifts in diversification rates across the insect tree of life, two shifts of which were characterized as major and associated to the origins of flight and of complete metamorphosis^26^. In a second study^23^, the authors compiled and analysed the insect fossil record at family-level and found out that the origin of wings is associated with an increase of both origination rates and extinction rates, whilst the development of complete metamorphosis yielded an increase in diversification rates. Nonetheless, a clear understanding of insects’ evolution is often more complex to figure out since conflicts between fossil and molecular inferences are known^28^ or because fossil data may be scarce for one particular group while phylogenetic data are extensive (e.g. Lepidoptera^11^,^25^) leading to spurious conclusions on the mode and tempo of diversification.

In this study, we first re-examine the global insect fossil record to account for the changes in family-level insect diversity^3^,^4^,^5^,^24^ and analytical methods^29^,^30^ that have occurred in the last twenty years. We then re-analyse a recently published molecular phylogenetic dataset using two distinct state-of-the-art diversification analysis methods, which either complement (TreePar^31^) or outperform (BAMM^32^) the earlier MEDUSA approach. Both the analyses of fossil and molecular data are expected to provide additional insights into the pattern of insect diversification over time, especially in relation to the role of *(i)* mass extinctions, *(ii)* environmental changes associated with the KTR and *(iii)* key innovations such as wings and complete metamorphosis.

## Results

### Inference of a global diversification pattern

After cleaning the fossil record, 38,013 and 39,240 occurrences were assembled for the family-level and the order-level fossil record, respectively (Supplementary Tables S1,2,S3).

**Figure 2:**
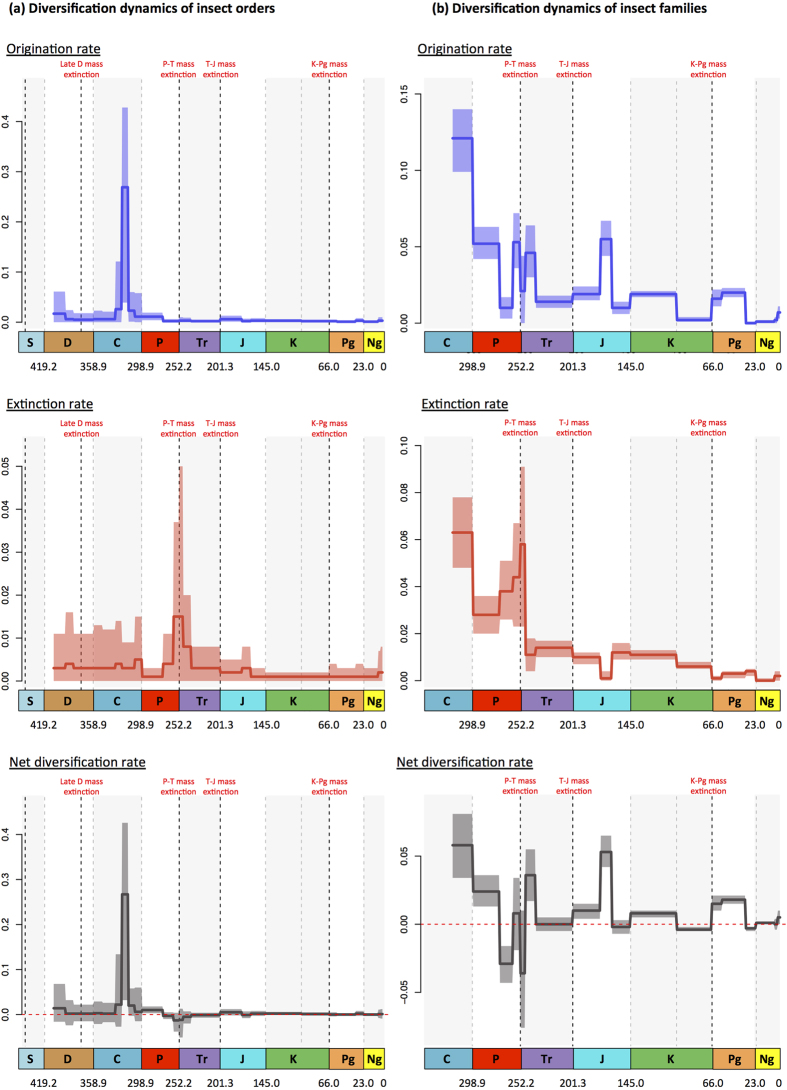
Global pattern of insect diversification based on the fossil record, which was analysed with a Bayesian model for the orders (**a**) and the families (**b**). Origination (blue) and extinction (red) rates were estimated using PyRate^29^,^30^ constrained with time bins as defined by epochs of the geologic timescale^60^. Solid lines indicate mean posterior rates, whereas the shaded areas show 95% HPD intervals. Net diversification rates (black) are defined as origination minus extinction. The vertical lines indicate the boundaries between geological boundaries and major mass extinction events. Abbreviations as in Fig. 1. Pictures and graphs made by Fabien Condamine. Figure drawn with PowerPoint.

We estimated speciation, extinction, and preservation rates using the Bayesian PyRate method to analyse the fossil record^29^,^30^. Analyses of the fossil orders show a burst of origination in the Late Carboniferous and a significant increase of extinction rate at the Permian-Triassic boundary. After the Triassic recovery, a fairly constant, near-zero diversification rate is estimated (no peak of origination or extinction is recovered, Fig. 2a). Except at the P-T event, we found no evidence for further impact of known mass extinctions such as the T-J and K-Pg events on ordinal diversification.

At the family level, we found an early burst of diversification in the Late Carboniferous and early Permian (Fig. 2b). Net diversification was primarily driven by high origination rates, outpacing extinction rates that were higher than all post-Palaeozoic intervals. Extinction rates increased beginning in the middle Permian, reaching a local peak in the Early Triassic. This peak represents the P-T mass extinction, although the middle Permian increase in extinction rates arises from artificial truncation of ranges due to incomplete sampling (the Signor-Lipps effect). The shift of peak extinction rates after the P-T boundary likely results from the PyRate method itself. Because the method estimates the true range (and therefore time of extinction) with a beta distribution, it will tend to infer extinction times that are too young when true ranges are instead abruptly truncated by a mass extinction.

Net diversification rates were low and fluctuated around zero during the post-Palaeozoic, except for significant diversification in the Middle Triassic, Middle Jurassic, and to a lesser extent the early Palaeogene. Early Palaeogene diversification may be artificially inflated by the presence of the exceptionally well-sampled Baltic amber (providing the oldest records of families that may have originated in the poorly-sampled Late Cretaceous) coupled with the ‘Pull of the Present’ bias in range-through data. Extinction rates notably decreased over post-Palaeozoic time, but we did not find a significant role of the Triassic-Jurassic or Cretaceous-Palaeogene extinction events. Late Cretaceous negative net diversification may in part reflect the K-Pg extinction, but could also be explained by mid-Cretaceous taxonomic turnover in the insect fauna, as recovered when the fossil record is analysed with time intervals of 10 Myrs (Supplementary Figure S1).

Phylogenetic analyses made with TreePar rejected the hypothesis of a rate-constant diversification within insects. Instead the best fitting-model – as indicated by LRT and AICc – is a model including five changes of diversification rates and turnover (Table 1). A model with one more shift did not significantly improve the likelihood. All corresponding changes of diversification rates correspond to minor downshifts, in which drops in net diversification rates are not marked. Interestingly, these shifts correspond to major changes of origination and/or extinction rates inferred with PyRate. Specifically, three of these downshifts were found in the Mesozoic: one at the end of the Early Jurassic (at 174 Myr ago), one in the Early Cretaceous (at 122 Myr ago), and one in the Late Cretaceous (at 70 Myr ago). The two other downshifts are inferred in the Cenozoic: one in the late Eocene (37 Myr ago), and one in the middle Miocene (14 Myr ago). We did not find any significant rate shifts in the Permian or Triassic. Although these five rate shifts did not correspond to any mass extinction event, the shift at 70 Myr ago is closely situated around the K-Pg mass extinction (66 Myr ago^33^), and the shift at 37 Myr ago is possibly consistent with the Eocene-Oligocene transition (33.9 Myr ago^34^), a pronounced cooling event. In addition, the results of the analyses in which the mass extinction option was set to TRUE also indicate that a constant birth-death is more likely than a model incorporating a mass extinction event (Supplementary Table S4). Finally, TreePar analyses suggest that the inferred turnover, which is the ratio of extinction rate over speciation rate, is very close to zero during all of insect evolutionary history (Table 1).

### Inference of speciation rate shifts, key innovations, and diversity-dependence

We used phylogenetic data coupled with the BAMM^32^ model, which is designed to automatically detect changes of speciation rates, to assess key innovation and diversity-dependence pattern. The BAMM analyses converged well as indicated by high ESS values (ESS_log-likelihood_ = 365.7, ESS_number of shifts_ = 494.2) and the stationarity of the MCMC (Supplementary Figure S2). We recovered eight significant rate shifts (Supplementary Figure S3) according to Bayes factors (BF = 94.98 over the null model). More specifically, the post burn-in posterior distribution of the number of shifts supported models with six shifts (PP < 0.02), seven shifts (PP = 0.24), eight shifts (PP = 0.36), nine shifts (PP = 0.24), ten shifts (PP = 0.09), and eleven shifts (PP = 0.04), respectively (Supplementary Figure S3). The 95% credible set of rate shift configurations sampled with BAMM also identified eight core shifts. The distinct shift configurations in the credible set (with the highest posterior probabilities) and the best configuration shift are both provided (Supplementary Figures S4,5).

**Figure 3:**
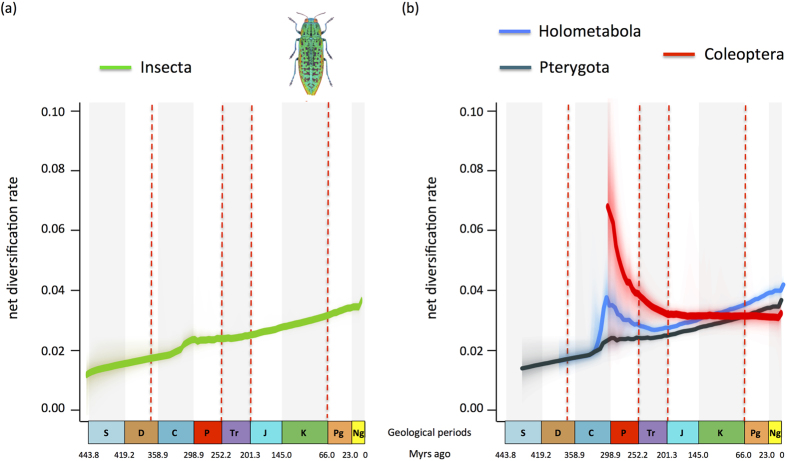
Global pattern of insect diversification based on a time-calibrated phylogeny. Diversification-through-time plots were computed for insect family (bottom left), and for the Pterygota, Holometabola and Coleoptera (bottom right). The corresponding plots illustrate the impact of the initial radiation of the Coleoptera on the diversification dynamics of Pterygota and Holometabola. Abbreviations as in Fig. 1. The drawing of extant jewel beetle and graphs were taken and made by Fabien Condamine and Gael Kergoat. Figure drawn with PowerPoint.

**Figure 4:**
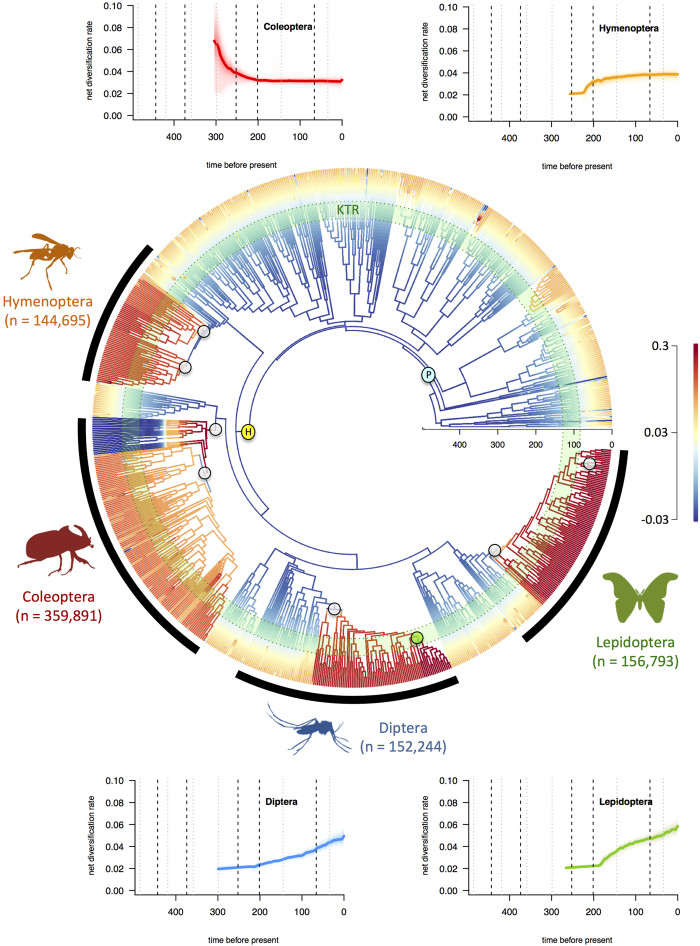
Phylogenetic pattern of insect macroevolution. Diversification shifts (inferred with BAMM) do not indicate an immediate impact on diversification for key innovations like Pterygota (P) and Holometabola (H). Instead shifts of diversification are located within the four richest insect orders; these shifts also predate the KTR, which is figured using a green circle. The eight shifts that are significantly supported are represented using small transparent circles; please not that the shift that occurred during the KTR is highlighted using a small green transparent circle. Diversification-through-time plots are depicted in each corner for the four most diverse insect orders. The phylogenetic tree, drawings (insect silhouettes), and graphs were taken and made by Fabien Condamine and Gael Kergoat. Figure drawn with PowerPoint.

The BAMM analyses suggest a global increase of diversification rate through time since the Devonian (Fig. 3a). The net diversification rates were never high, ranging from 0.015 events/Myr/lineage in the Devonian to 0.035 events/Myr/lineage in the Cenozoic. A notable change of diversification occurred around 300 Myr ago. All eight significantly supported shifts clearly postdate the origin of flight or complete metamorphosis (Fig. 4). Only one of these shifts (in a clade of Diptera; highlighted using a small transparent green circle in Fig. 4) occurred during the KTR. Seven of these shifts were recovered within the holometabolous clade containing the richest orders: Coleoptera (beetles, ~360,000 species), Diptera (flies and mosquitoes, ~152,000 species), Hymenoptera (ants, bees and wasps, ~145,000 species), Lepidoptera (butterflies and moths, ~157,000 species). Those rate shifts either occurred shortly after the origin of the group – in the case of beetles – or tens of millions years later-in the case of the three other orders (Fig. 4). One of these major changes in diversification dynamics corresponds to the initial radiation of the most basal coleopteran lineages (Archostemata, Adephaga and Myxophaga), which represents the main diversification shift that is detectable when analysing either the Pterygota or the Holometabola separately (Fig. 3b). A last shift is also inferred shortly before the origination of the clade grouping the Coleoptera and the Strepsiptera (Fig. 4). The results of BAMM analyses thus do not support the hypothesis that the two considered key innovations (flight and complete metamorphosis) generated a change in diversification dynamics (Fig. 3b, Supplementary Figures S6–8). We hypothesize that other clade-specific key innovations better explain the inferred macroevolutionary pattern. Further analyses to test for specific key innovations would be ideal to confirm that hypothesis. Birth-death models linking a particular trait (e.g. winged *versus* flightless, or holometabolous *versus* non-holometabolous) with speciation and/or extinction rates are available^35^. Nonetheless, this approach is currently not or hardly doable because of two main limitations: *(i)* large species-level phylogenies with a dense sampling (i.e. more than 80% of the extant diversity) are not available for groups encompassing both winged and flightless species, and *(ii)* diversification methods that allow testing a link between trait and diversification rates are currently seriously questioned^36^.

The BAMM analyses also allowed us to infer the clade-specific diversification pattern of the four richest insect orders (Fig. 4). Interestingly, the macroevolution pattern inferred for Coleoptera suggests an early period of fast diversification (0.08 events/Myr/lineage) between their origin and the P-T event, followed by a drastic slowdown of diversification rate until the Cenozoic (0.04 events/Myr/lineage). By contrast, the three other hyperdiverse orders (Diptera, Hymenoptera, and Lepidoptera) share a similar macroevolutionary pattern characterized by increases of diversification rate through time, i.e. starting from low rates (0.02 events/Myr/lineage in the Mesozoic or Palaeozoic) and trending towards higher rates (0.04 [Hymenoptera] or 0.06 [Diptera and Lepidoptera] events/Myr/lineage).

## Discussion

What gave rise to the insects’ staggering biodiversity? This question has fascinated many evolutionary biologists for centuries^5^,^6^. Although insect fossil and phylogenetic data have been accumulated at a slower rate compared to vertebrate or plant clades, they have nonetheless allowed investigation of several major explanatory hypotheses^1^,^3^,^4^,^5^,^6^. The most common explanations propose that intrinsic features of insects such as their small size, the presence of an exoskeleton, or the development of new key-innovations (wings, complete metamorphosis, specialized mouthparts, parasitoid habit …) best explain the long-lasting evolutionary success of insects. On the other hand, environmental and physical factors like climate/geological changes or the apparition of new ecological niches may also constitute evolutionary drivers, generating drastic and often sudden adaptive responses in insects. Our study attempts to bring new insights to progress in our understanding of insect diversification with an assessment of the fossil record and phylogenetic tree at the family level.

We first assessed the possible impacts of known past mass extinctions on insect diversification. Fossil-based diversification estimates demonstrate the impact of the P-T mass extinction as a key event in the evolutionary history of insects at both the ordinal and familial levels (Fig. 2)^37^. In contrast to the pronounced extinction revealed by fossil data, the TreePar phylogenetic analyses did not indicate a shift in diversification rate at the P-T boundary. As underlined by a study based on phylogenetic supertrees^38^, we cannot exclude the hypothesis that more extant orders ranged back to the Permian and survived the P-T mass extinction. However, it is more likely that the phylogeny used in TreePar analyses, which only contains extant taxa, has little power to resolve diversification rate changes when nearly all families are now extinct, as was the case in the Permian.

Interestingly, while TreePar supported a macroevolutionary scenario with five downshifts of diversification, only a single shift 70 Myr ago potentially corresponds to a mass extinction (the K-Pg event). In contrast, aside from slightly negative net diversification during the Late Cretaceous, there is little evidence from the fossil record for a significant K-Pg mass extinction of insects. However, the Late Cretaceous insect record is sparse, especially in the latest Cretaceous Maastrichtian stage, hindering recognition of a mass extinction. Negative net diversification could partially arise from the K-Pg extinction and is consistent with the downshift of diversification from TreePar. A second shift, the shift at 37 Myr ago, corresponds closely to a lesser mass extinction that occurred at the late Eocene-Oligocene transition, which is also evidenced with fossil data showing a negative net diversification in the Oligocene (Fig. 2).

Nonetheless, TreePar analyses did not significantly support a model incorporating mass extinction events over a model following a constant birth-death process and there is little evidence from the fossil record for extinctions other than the P-T event. Furthermore, the fact that the inferred turnover is very close to zero is consistent with a global diversification pattern mostly driven by low speciation rates and extinction rates that are close to zero, in agreement with the conclusions drawn from the analyses of the fossil record (extinction rates range between 0.0 and 0.1 at the P-T event). All of this provides further evidence to support the hypothesis that insects were quite impervious to past mass extinctions events, except during the late Permian (which was only detectable with fossil data) and possibly the Late Cretaceous (found in TreePar). This hypothesis is supported by other works including fossil^1^,^4^,^39^ and phylogenetic^14^,^38^ studies.

Our analyses also bring insight into the role of main key innovations, such as the origin of flight or the evolution of complete metamorphosis, on insect diversification. Here we present and discuss the pattern referred to as the ‘mean phylorate’, which summarizes all rate shifts recovered with BAMM in a way similar to a consensus phylogeny with polytomies. It is worth underlining that we cannot pin down the precise locations of these shifts because: *(i)* multiple rate shift configurations can explain the data; and *(ii)* rate shifts are not independent. Based on the marginal shift probabilities, the approximate location of the eight statistically supported rates shifts can be determined (Fig. 4, Supplementary Figure S4). Contrary to the MEDUSA inferences made on insects^26^, BAMM analyses did not recover significant shifts in diversification rate associated with the origin of flight (in Pterygota) or complete metamorphosis (in Holometabola) (Figs 3b,4). Instead, seven out of eight shifts of diversification regimes are found within the four richest insect orders, but not at the origin of key innovations (Fig. 4 and Supplementary Figure S4). In contrast, fossil-based analyses showed high net diversification rate in the Late Carboniferous, possibly associated with the appearance of the first winged insects in the fossil record (Fig. 2). This early burst of diversification would be consistent with the importance of flight as a key innovation, assuming the rapid appearance of fossil insects in the mid-Carboniferous is a true pattern rather than an artefact of changing fossil preservation conditions (i.e. an incomplete fossil record). Phylogenetic analyses avoid problems of a potentially incomplete fossil record and suggest that the evolution of flight did not cause a shift in the diversification rate. However, the phylogeny contains very few lineages in the early history of insects, a time in which virtually all richness belonged to extinct groups. Interestingly, both the phylogeny and the fossils provide good evidence that the complete metamorphosis was not an important key innovation (Fig. 4, Supplementary Figure S8). Insects diversified rapidly early in their history, but virtually none of that early diversification was among Holometabola, which increased gradually and slowly in prominence over time. An observer in the Permian period might have asked the same question about key innovations in Polyneoptera, because that clade was the most diverse and Holometabola was quite rare.

In Coleoptera, major shifts of diversification rates occurred before and shortly after the appearance of the group (Supplementary Figure S4), resulting in a major slowdown of diversification rates (Fig. 3b), a pattern fitting one of the expectations under a diversity-dependence process^40^,^41^. Interestingly, the exact same diversification dynamic was recovered in a study focusing on the beetle fossil record^39^, which also evidenced the subsequent decline of the Adephaga, Archostemata and Myxophaga (Fig. 3). Here, we may hypothesize that the development of elytra and the subsequent ecological radiation of the Polyphaga likely played a major role in coleopteran diversification. Further studies may focus on the initial radiation of beetles and investigate the pattern of early rapid diversification potentially attributed to an adaptive radiation^6^,^42^. Similarly, the shifts within Diptera and Hymenoptera may be consistent with the development of trophically specialized habits (i.e. parasitoid) or the development of specialized mouthparts and of the associated feeding specialization in Diptera^1^. Finally the shifts within Lepidoptera are likely associated with the radiation of the Apoditrysia, which include mostly larger moths and the butterflies. A first shift occurred *ca.* 85 Myr after the origination of Lepidoptera (*ca.* 270 Myr ago), whereas a second shift (*ca.* 50 Myr ago) coincides with the radiation of quadrifid noctuoids, a clade that is characterized by the development of a tympanic organ that serves as a defence against bat predators^43^. Interestingly, all inferred diversification rate shifts, but one, either largely predate (six shifts out of eight) or postdate (the second shift in Lepidoptera) the KTR (Fig. 3), suggesting that the rise of Angiosperms did not generate an immediate increase in insect diversification within major insect groups. The Bayesian fossil-based analyses also did not evidence an obvious increase of net diversification in the Cretaceous as expected (Fig. 2b). However we cannot completely rule out the influence of the radiation of Angiosperms on insect diversification. First, a BAMM shift was inferred *ca.* 95 Myr ago in a diverse dipteran clade that encompasses several phytophagous families such as the Agromyzidae or the Anthomyiidae. Second, a global increase of diversification after 80 Myr ago and throughout the Cenozoic is noticeable on the mean phylorate tree (Fig. 4), although such an increase is not supported by the fossil data. This is consistent with the hypothesis that diversification among extant families may have accompanied Angiosperm radiation and expansion^1^,^6^,^7^,^42^. This is particularly true for some groups, like the Apoditrysia, a pattern that has already been proposed^11^,^22^ (Fig. 4). If true, that radiation must have been offset by extinctions among more basal groups to yield little net diversification, as shown by the fossil data. The fact that we do not support the KTR as a trigger of insect diversity may be explained if angiosperm-pollinating insects first diversified on gymnosperm plant hosts, before initially shifting after the KTR with generalist angiosperm associations and later to specialist associations during the Late Cretaceous and Paleogene, as the extant radiation we see today^23^,^44^,^45^. Initial evolution of feeding strategies and host associations may also be decoupled from taxonomic diversification within those ecological categories. Alternatively, fossil data indicate that the P-T and K-Pg extinctions had a devastating effect on plant-insect associations^44^, resulting in major lags in plant-host recovery and their subsequent colonization by insect herbivores^9^. Thus extinction events may have erased the signal of lineage radiation during the KTR.

The results of BAMM analyses may be compared to the results of MEDUSA analyses relying on the same insect phylogeny^26^. In contrast with MEDUSA analyses, we found only eight significant rate shifts instead of 45. This high level of discrepancy may result from the fact that both approaches have fundamental design differences. Notably the BAMM^32^ model, contrary to MEDUSA^27^, is explicitly designed to account for variation in evolutionary rates over time^46^ and among lineages (MEDUSA only accounts for clade variation^27^). BAMM therefore outperforms MEDUSA for tested scenarios with time-varying rates of species diversification^32^; in this context BAMM also better estimates the correct number of processes and branch-specific estimates of speciation rates^32^. More concerns have been raised about MEDUSA with a recent simulation study^47^ showing that MEDUSA has an extremely high type I error rate, which can result in spurious diversification shifts and provides severely biased parameter estimates of diversification. Taken altogether, these studies cast doubts on the results and conclusions drawn from MEDUSA analyses. It is also important to correctly interpret results from BAMM analyses. As underlined by the author, BAMM analyses identify distinct sets of shifts that are sampled together, and further compute their relative probabilities^32^.

Our assessment and analysis of the fossil record and the analysis of a densely sampled family-level phylogeny provide further evidence supporting the hypothesis that modern insect diversity does not result from high speciation rates but rather from an ancient origin associated with low extinction that sustained diversification in a variety of niches as they appeared. Phylogenetic results imply that the radiation of the four richest holometabolous orders was instrumental in shaping the insect diversification dynamics, although the fossil data suggest a pronounced early radiation but low diversification rates as Holometabola rose dominance. Because major increases of diversification postdate the development of wings and complete metamorphosis or predate the origin and main period of angiosperm radiation, other factors must be investigated to explain the global pattern of insect diversification (e.g. clade-specific innovations, contrasted responses to past environmental changes).

Our results and conclusions about the global diversification pattern of insects depend upon the quality of the underlying data (both fossil and phylogenetic). The effect of incomplete taxon sampling, inaccurate divergence time estimations and small-sized phylogenies are potentially serious problems that are difficult to address. For instance, we were unable to perform our diversification analyses over a sample of dated trees because we had no access to the posterior distribution of the dating analyses of Ref. 26. This can lead to inaccurate parameter estimates or wrong model selections. We also do not think BAMM is free of potential methodological issues especially because BAMM assumes an extinction rate-constancy through time, which may explain some differences with other models (see the macroevolutionary inference for the Cetacea^32^,^46^). These potential analytical limitations may constrain the certainty of our interpretations and conclusions. Yet, as long as the fundamental hypothesis-testing nature of the used analyses is kept in mind, they still remain our best window into understanding the rich, deep past of Earth’s stupendous biological diversity. We hope that this study will provide interesting perspectives for future investigations on other model groups (e.g. Mollusca), which have both suitable fossil and phylogenetic data for higher-level taxonomy.

## Methods

### Family-level taxon sampling

As current phylogenetic trees are incomplete at species-level for insects, we followed the views of other authors and focused on studying their diversification pattern at the family level^1^,^4^,^24^. This taxonomic level has been analysed in other studies of fossil diversity and appears to correlate well with underlying species diversity^3^,^24^,^25^. Families are less susceptible to irregular and biased sampling than fossil species and genera, hence evolutionary signals are stronger and better maintained at this level. Insect families, especially extant ones, are also reasonably well established through consensus among researchers, whereas fossil species and genera are more idiosyncratically defined; they less frequently correspond to good phylogenetic units (i.e. monophyletic groups). Insect families individually possess discrete, often highly stereotyped life habits, and their morphologies directly reflect their trophic guilds, which are informative in diversity studies.

### Fossil and phylogenetic data

Fossil occurrence data were downloaded on a recent version of the *Paleobiology Database* by selecting the ‘*family level*’, ‘*order level’*, and ‘*geological epochs*’ options (last accessed October 15^th^, 2015), mostly compiled by M.E.C. We downloaded 40,046 fossil occurrences, and cleaned the data by checking for synonymies among all taxa. The final dataset contains 38,013 and 39,240 occurrences for the family-level and the order-level fossil record, respectively. The two datasets are available on the website of *Scientific Reports* (Supplementary Tables S1,2).

Phylogeny-based diversification analyses were carried out using a comprehensive insect timetree^26^ encompassing 874 terminal taxa representing 82% of the extant insect families. The corresponding phylogeny was reconstructed with eight genes (three mitochondrial and five nuclear genes) using maximum likelihood phylogenetic inference (see Ref. 26 for more details). The dating analyses were carried out within a Bayesian framework under an uncorrelated relaxed-clock model calibrated with 89 fossil calibrations, and using the maximum likelihood tree as a starting tree^26^.

### Phylogeny-based diversification analyses

Diversification rates can vary over the evolutionary history of a clade, for example in response to changes in the biotic and abiotic environments, diversity-dependent effects, or the combination of both. A straightforward and widespread approach to account for time variation in diversification rates is to assume a functional dependence of speciation and extinction rates with time^48^. Likelihood expressions of phylogenetic branching times for such models are available, for both continuous (e.g. exponential variation^32^,^46^) and discrete (referred to as the ‘discrete shift’ model^31^) forms of time variation. These time-dependent models allow a quantitative estimation of how diversification rates have varied through time.

### Episodic time-variation of diversification

The TreePar^31^ method was used in our study to test for the presence of global diversification changes, and the effect of mass extinctions identified in the fossil record. This method assesses global speciation and extinction rates, and their respective variations through time by relaxing the assumption of constant rates and allowing rates to change at specific points in time. Such a model allows for the detection of rapid changes in speciation and extinction rates due to environmental factors like the geological changes or mass extinction. We employed the ‘*bd.shifts.optim*’ function that allows for estimating discrete changes in speciation and extinction rates and mass extinction events in under-sampled phylogenies. Going backward in time, it estimates the maximum likelihood speciation and extinction rates together with the rate shift times *t* = (t_1_, t_2_, …, t_n_) in a phylogeny. At each time *t*, the rates are allowed to change and the species may undergo a shift in diversification. TreePar analyses were run as follows: start = 0 (present), end = insect crown age (479 Myr ago^26^), grid = 1 Myr (examining potential rate shifts every 1 Myr), and posdiv set to FALSE to allow the diversification rate to be negative (periods of declining diversity). By default the option mass extinction (ME) was set to FALSE, but additional analyses were also ran setting the option mass extinction in the model, to specifically test the presence of such event shaping the phylogenetic tree of insects.

### Across-clade and time-variation diversification

We used the Bayesian Analysis of Macroevolutionary Mixture (BAMM) to estimate speciation and extinction rates through time and among/within clades^32^,^49^,^50^. BAMM is an analytical tool for studying complex evolutionary processes on phylogenetic trees, potentially shaped by a heterogeneous mixture of distinct evolutionary dynamics of speciation and extinction across clades. The method uses reversible jump MCMC (rjMCMC) to automatically detect rate shifts and sample distinct evolutionary dynamics that best explain the whole diversification dynamics of the clade. Within a given regime, evolutionary dynamics can involve time-variable diversification rates; in BAMM, the speciation rate is allowed to vary exponentially through time while extinction is maintained constant^32^,^50^. Subclades in the tree might diversify faster (or slower) than others, and BAMM allows detecting these diversification rate shifts and compare how many and where these shifts might occur. BAMM provides estimates of marginal probability of speciation and extinction rates at any point in time along any branch of the tree. Marginal probabilities of the number of evolutionary regimes can also be computed, allowing comparisons of models with a given number of shifts with Bayes factors.

BAMM is implemented in a C^++^ command line program and the BAMMtools R-package^51^. We set four rjMCMC running for 50 million generations and sampled every 50,000 generations. BAMM handles random incomplete taxon sampling using the implemented analytical correction, but it was not possible to use this sampling fraction taking into account the missing species here since the insect tree only contains 1% of the total diversity. Instead, we incorporated information on species richness for each family in the form of branch-specific sampling fractions (Supplementary Table S5). Other settings were set to default except the Poisson process prior that we set to 0.5 following the author’s recommendation. We performed four independent runs (with a burn-in of 15%) using different seeds, and we used effective sample size (ESS) to assess the convergence of the runs, considering values above 200 as indicating good convergence. The posterior distribution was used to compute the mean global rates of diversification through time (‘mean phylorate’), to estimate the configuration of the diversification rate shifts by evaluating alternative diversification models as compared by Bayes factors, and get clade-specific rates through time when a distinct macroevolutionary regime is identified.

### Differences between TreePar and BAMM

It is worth underlining that TreePar and BAMM constitute very distinctive methods that differ in several points on the way speciation and extinction rates are estimated. As mentioned beforehand both approaches are used here to assess distinct patterns: TreePar assesses global patterns and is used here to investigate extinction while BAMM is used to automatically detect rate shifts within and among clades. Thus our diversification analyses should be seen as complementary but not as cross-validation procedure.

We see at least four main differences between the two approaches: *(i)* TreePar allows extinction to vary over time while BAMM assumes extinction is constant over time; *(ii)* TreePar estimates global and sudden diversification shifts over time (potentially due to mass extinctions or a response to environmental changes), and does not distinguish clade-wise shifts. On the contrary, BAMM estimates clade-wise shifts of diversification coupled to time-dependence variation of rates. Here we used TreePar to estimate speciation and extinction rates during a specific time interval (i.e. 50 Myr ago until the crown age of insects at 479 Myr ago), whereas BAMM was used to examine the whole clade evolutionary history; *(iii)* TreePar takes individual branch as a single lineage, whereas we informed each lineage with the total clade diversity for BAMM analyses; and *(iv)* both methods also differ in the way they deal with incomplete taxon sampling. TreePar allows the user to specify the fraction of sampled diversity in the dataset while BAMM allows the user to incorporate information on species richness for each sampled lineage.

### Model selection

For TreePar approach, we computed the corrected Akaike information criterion (AICc) based on the log-likelihood and the number of parameters of each diversification model^52^. A difference of two in the AICc (ΔAIC) between two models suggests significant support for the model with the lowest AICc. Akaike weights (ωAIC) were then computed based on AICc. We also checked support for the selected model against all models nested within it using the likelihood ratio test (LRT), which shows significant support at *P* < 0.05. The scenario supported by LRT and with the lowest AICc was considered the best. If the model with the lowest AICc was not supported by LRT, the model with less parameters was considered the best. For the BAMM analyses, we used posterior probabilities and Bayes factors to compare the fit of different evolutionary scenarios (i.e. with no shift, one shift …). We considered Bayes factors values above 6 to significantly favour one model over another^53^. BAMMtools makes it easy to compute Bayes factor evidence in favour of one model relative to another. To do so we used the ‘*computeBayesFactors*’ function applying a burn-in of 15%. As explained on the BAMM website, it is very important to recognize that model probabilities for rarely sampled models are likely to be inaccurate.

### Fossil-based diversification analyses

Our purpose was to stress the global diversification dynamics, but not the species diversity dynamics as Labandeira and Sepkoski^1^ did twenty years earlier.

Analysing the fossil record is not a trivial task, in part because the fossil record is biased or incomplete. Compensating for these biases may be challenging because fossil preservation varies with time and space^54^. We used a Bayesian model that simultaneously infers the temporal dynamics of origination and extinction, as well as the preservation^29^. This approach, implemented in the program PyRate^30^ 0.600 (https://github.com/dsilvestro/PyRate, last accessed October 15, 2015), uses as input data all fossil occurrences that can be assigned to a taxon, in this case fossil genera, to jointly model the preservation and diversification processes. The preservation process infers the individual origination and extinction times of each taxon based on all fossil occurrences and on an estimated preservation rate, denoted *q*, and expressed as expected occurrences per taxon per Myr.

We followed the PyRate approach as the study that analysed the evolutionary history of vascular plants^55^. This approach included appropriate modifications for the fossil data and the aims of this study, which is focused on variation in origination and extinction at global scale and large temporal ranges. We used here a homogeneous Poisson process (*-mHPP* option) of preservation^29^. We also accounted for varying preservation rates across taxa using the Gamma model (*-mG* option), that is, with gamma-distributed rate heterogeneity^29^. Because of the large number of occurrences analysed and of the vast timescale considered, we used here eight rate categories to discretize the gamma distribution (*-ncat 8* option), to accommodate the potential for more variability of preservation rates across taxa.

Furthermore, we dissected the birth–death process into time intervals (*-fixShift* option), defined by the geological periods of the stratigraphic timescale, and estimated origination and extinction rates within these intervals (*-A 0* option)^55^. We adopted this solution as an alternative to the algorithms implemented by default in PyRate that jointly estimate the number of rate shifts and the times at which origination and extinction undergo a shift (*-A 2* option)^29^,^30^. The estimation of origination and extinction rates within fixed time intervals improved the mixing of the MCMC and allowed us to obtain an overview of the general trends of rate variation throughout a long timescale^55^. Both the preservation and the birth–death processes are still modelled in continuous time and are not based on boundary crossings. Thus, the origination and extinction rates are measured as the expected number of origination and extinction events per lineage per Myr. A potential issue in fixing *a priori* the number of rate shifts is over-parameterization. We overcame this issue by assuming that the rates of origination and extinction are part of two families of parameters following a common prior distribution, with parameters estimated from the data using hyper-priors^55^.

We ran PyRate for 5 millions of MCMC generations on each of the 10 randomly replicated datasets (using the *extract.ages* function, with *replicates = 10*). After excluding the first 20% of the samples as burn-in period, we combined the posterior estimates of the origination and extinction rates across all replicates to generate rates-through-time plots (origination, extinction, and net diversification). Rates of two adjacent intervals were considered significantly different when the mean of one lay outside of the 95% HPD of the other, and conversely. We looked at the marginal posterior distributions of origination and extinction rates through the largest extinction events documented in geological history, the mass extinction events in life’s history. In particular, we examined the diversification dynamics at the Late Devonian, the Permian-Triassic, the Triassic-Jurassic, and the Cretaceous-Paleogene mass extinctions. We focused on the magnitude of rate changes, their statistical significance and the uncertainty around those estimates. We conducted the analyses twice: one for the family-level diversity and one for the order-level diversity as one might expect family extinctions or order extinctions.

## Additional Information

**How to cite this article**: Condamine, F. L. *et al.* Global patterns of insect diversification: towards a reconciliation of fossil and molecular evidence? *Sci. Rep.* **6**, 19208; doi: 10.1038/srep19208 (2016).

## Supplementary Material

Supplementary Table S1

Supplementary Table S2

Supplementary Table S3

Supplementary Information

## Figures and Tables

**Figure 1:**
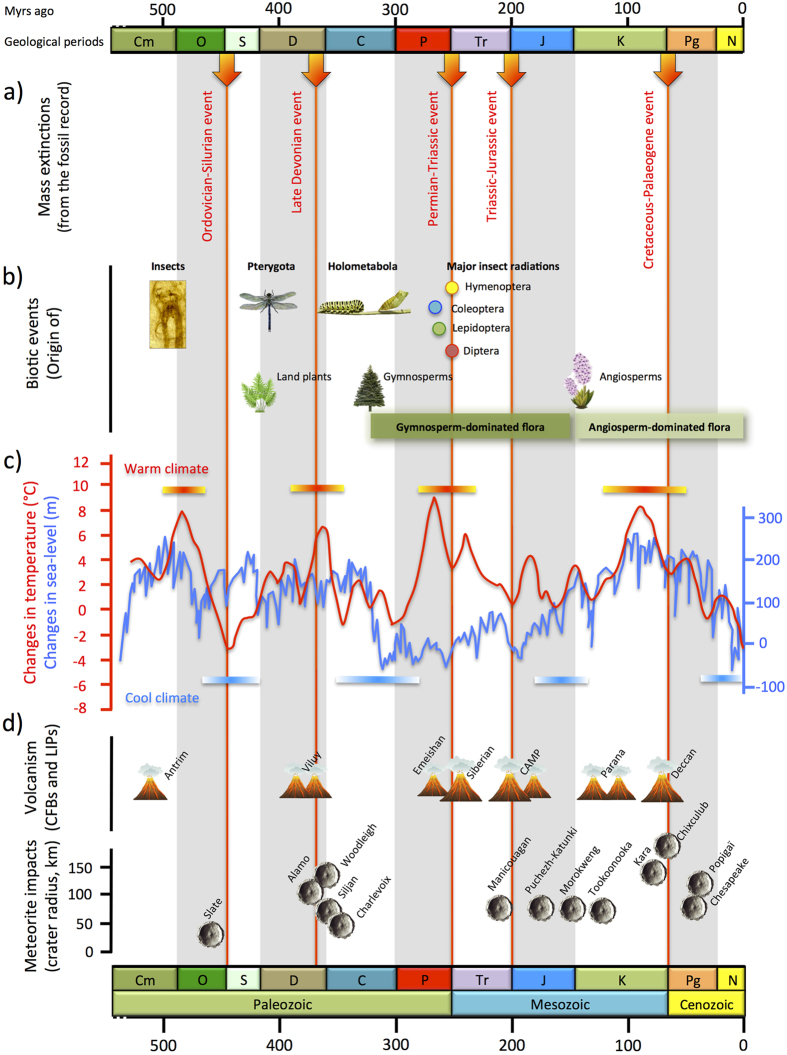
An overview of the possible triggers of insect diversification over time. The insect evolution was punctuated by (**a**) five known mass extinction events (red arrows), and has seen the (**b**) origin of main key innovations and the rise of major clades. Environmental changes may also be major determinants of diversity dynamics like the (**c**) global temperature (red curve highlighting the cooling and warming events) and as sea-level fluctuations (blue curve); as well as (**d**) geological events such as volcanism due to tectonic movements (CFBs, continental flood basalts; LIPs, large igneous provinces), or meteorite impacts, which modify atmosphere composition and impact diversity. Cm = Cambrian, O = Ordovician, S = Silurian, D = Devonian, C = Carboniferous, P = Permian, Tr = Triassic, J = Jurassic, K = Cretaceous, Pg = Paleogene, and N = Neogene. The pictures (fossil and tree), drawings (dragonfly, caterpillar, and plants), and graphs were taken and made by Fabien Condamine. Figure drawn with PowerPoint.

**Table 1:** Results of TreePar diversification analyses.

Model	No shift time	1 shift time	2 shift times	3 shift times	4 shift times	5 shift times	6 shift times
**NP**	2	5	8	11	14	**17**	20
**logL**	-4627.4655	-4460.5042	-4417.4111	-4408.6636	-4403.0704	**-4398.6822**	-4396.4364
***P*** **(LRT)**	null model	≪0.0001	≪0.0001	0.0006	0.0108	**0.032**	0.213
**AICc**	9258.945	8931.078	8850.989	8839.633	8834.63	**8832.079**	8833.858
**∆AIC**	426.866	98.999	18.910	7.554	2.551	**0**	1.779
**ωAIC**	0	0	0	0.013	0.163	**0.584**	0.24
**r1**	0.0074	6,00E-04	6,00E-04	6,00E-04	6,00E-04	**1,00E-04**	1,00E-04
**τ1**	0	0.0028	0.0028	0.0024	0.003	**0.0046**	0.0042
**st1**	—	37	37	37	37	**14**	14
**r2**	—	0.0094	0.0068	0.0051	0.0051	**9,00E-04**	9,00E-04
**τ2**	—	0	0	0.0013	0	**0.0031**	0.0043
**st2**	—	—	122	70	70	**37**	37
**r3**	—	—	0.0128	0.0081	0.0082	**0.0051**	0.0051
**τ3**	—	—	0	5,00E-04	0	**0.0037**	0.0068
**st3**	—	—	—	122	122	**70**	70
**r4**	—	—	—	0.0128	0.0108	**0.0081**	0.0082
**τ4**	—	—	—	0	0.001	**0.0043**	0.0027
**st4**	—	—	—	—	174	**122**	122
**r5**	—	—	—	—	0.0145	**0.0108**	0.0114
**τ5**	—	—	—	—	0.0014	**0.0012**	0.0037
**st5**	—	—	—	—	—	**174**	165
**r6**	—	—	—	—	—	**0.0145**	0.0071
**τ6**	—	—	—	—	—	**0.0036**	0.0035
**st6**	—	—	—	—	—	—	174
**r7**	—	—	—	—	—	—	0.0146
**τ7**	—	—	—	—	—	—	0

The model with five shifts is supported. Abbreviations: NP, number of parameters; logL, log-likelihood; *P* (LRT), p-value of the likelihood ratio test (LRT are realized sequentially by testing first the null model with the second model; if the second model receives a significant support it becomes the reference for the second LRT against the third model, and so on); AICc, corrected Akaike Information Criterion; ∆AIC, difference in AIC between a model and the best model; ωAIC, Akaike weight; r, rate of diversification; τ, turnover (extinction/speciation); st, shift time, in which ‘r1′ denotes the diversification rate and ‘τ1′ is the turnover, both inferred between Present and the shift time 1 (‘st1′).
