# Supplementary material for: Global patterns of insect diversification: towards a reconciliation of fossil and molecular evidence?
Source: Sci Rep. 2016 Jan 18;6:19208. doi: 10.1038/srep19208 (PMC4725974; doi:10.1038/srep19208)

Supplementary Information

Global patterns of insect diversification: towards a reconciliation of fossil and molecular evidence?

Fabien L. Condamine, Matthew E. Clapham & Gael J. Kergoat

<u>**Contents**</u>

Table S1. Fossil occurrences of insect orders from Paleobiology Database.

Table S2. Fossil occurrences of insect families from Paleobiology Database.

Table S3. Complete reference list of the original download.

Table S4. Results of TreePar diversification analyses with mass extinction.

Table S5. Species richness within extant insect families.

Figure S1. Global pattern of insect diversification based on the fossil record for the orders (a) and the families (b).

Figure S2. Convergence of the BAMM analysis (a, stationary of the Markov chain Monte Carlo; b, sampling of shifts along the Bayesian run).

Figure S3. Frequency distribution of distinct macroevolutionary rate regimes estimated using BAMM (a, prior distribution of shift; b, posterior distribution of shifts).

Figure S4. Credible set of configuration shifts of insects inferred with BAMM.

Figure S5. The best shift configuration inferred with BAMM for insects.

Figure S6. Global pattern of diversification rates over the insect evolution (top, speciation rate; middle, extinction rate; bottom, net diversification rate)

Figure S7. Pattern of diversification rates for the five richest insect orders and the rest of insect (curves represent the net diversification rates).

Figure S8. Contrasting the diversification rates of non-Pterygota versus Pterygota (top plots), and the diversification rates of non-Holometabola versus Holometabola (bottom plots). One can see that no significant increase of diversification is detected at the origin of Pterygota, nor at the origin of Holometabola.

Table S1. Fossil occurrences of insect orders from Paleobiology Database.

Table S2. Fossil occurrences of insect families from Paleobiology Database.

Table S3. Complete reference list of the original download.

The tables are given in separate files that are available on the website of *Scientific Reports* (or are available upon request to F.L.C.).

Table S4. Results of TreePar diversification analyses with mass extinction. The model with constant diversification is supported. Abbreviations: NP, number o parameters; logL, log-likelihood; *P* (LRT), p-value of the likelihood ratio test (LRT are realized sequentially by testing first the null model with the secon model; if the second model receives a significant support it becomes the reference for the second LRT against the third model, and so on); AICc, correcte Akaike Information Criterion; ΔAIC, difference in AIC between a model and the best model; ωAIC, Akaike weight; Net. div. rate, net diversification rat (speciation minus extinction); Turnover, ration extinction over speciation; sp, survival probability (i.e. sp = 1 means that at time *i*, a rate shift may occur but n species go extinct); ME, shift time of mass extinction, in which 'ME1' denotes the time of the first mass extinction.

Model	Constant BD	Model with one ME	Model with two ME	Model with three ME
**NP**	**2**	4	6	8
**logL**	**-4607.471**	-4607.4709	-4607.4709	-4607.4709
***P* (LRT)**	**null model**	0.99	0.99	0.99
**AICc**	**9218.956**	9222.988	9227.039	9231.108
**ΔAIC**	**0**	4.03	8.08	12.15
**ωAIC**	**0.867**	0.116	0.015	0.002
**Net div. rate**	**0.008**	0.008	0.008	0.008
**Turnover**	**0**	0	0	0
**sp1**	**-**	1	1	1
**ME1**	**-**	464	464	464
**sp2**	**-**	-	1	1
**ME2**	**-**	-	466	465
**sp3**	**-**	-	-	1
**ME3**	**-**	-	-	466

Table S5. Species richness within extant insect families. The table shows for each tip in the tree analysed in this study to which higher level taxon it belongs, the family name, the current number of species described in the family, and the branch-specific sampling fraction used for the BAMM analyses.

Tip label	Higher level taxon	Family name	Species richness	Branch-specific sampling fraction (BAMM)
Archaeogna	Archaeognatha	Archaeognatha	495	0.00202020202020202
Bla_Blaber	Blattodea	Blaberidae	1198	0.000834724540901503
Bla_Blatti	Blattodea	Ectobiidae	2381	0.000419991600167997
Bla_Crypto	Blattodea	Blattidae	594	0.00168350168350168
Bla_Ectobi	Blattodea	Cryptocercidae	12	0.0833333333333333
Bla_Noctic	Blattodea	Nocticolidae	32	0.03125
Bla_Polyph	Blattodea	Corydiidae	215	0.00465116279069767
Co_Ad_Amph	Coleoptera	Amphizoidae	5	0.2
Co_Ad_Aspi	Coleoptera	Aspidytidae	2	0.5
Co_Ad_Cara	Coleoptera	Carabidae	40000	0.000025
Co_Ad_Dyti	Coleoptera	Dytiscidae	4015	0.00024906600249066
Co_Ad_Gyri	Coleoptera	Gyrinidae	882	0.00113378684807256
Co_Ad_Hali	Coleoptera	Haliplidae	218	0.00458715596330275
Co_Ad_Hygr	Coleoptera	Hygrobiidae	5	0.2
Co_Ad_Note	Coleoptera	Noteridae	250	0.004
Co_Ad_Trac	Coleoptera	Trachypachidae	6	0.166666666666667
Co_Ar_Cupe	Coleoptera	Cupedidae	31	0.032258064516129
Co_Ar_Micr	Coleoptera	Micromalthidae	1	1
Co_Ar_Omma	Coleoptera	Ommatidae	6	0.166666666666667
Co_Myx_Lep	Coleoptera	Lepiceridae	1	1
Co_Myx_Hyd	Coleoptera	Hydroscaphidae	22	0.0454545454545455
Co_Myx_Sph	Coleoptera	Sphaeriusidae	19	0.0526315789473684
Co_Myx_Tor	Coleoptera	Torridincolidae	60	0.0166666666666667
Co_P_Aderi	Coleoptera	Aderidae	900	0.00111111111111111
Co_P_Agyrt	Coleoptera	Agyrtidae	70	0.0142857142857143
Co_P_Alexi	Coleoptera	Alexiidae	50	0.02
Co_P_Anobi	Coleoptera	Anobiidae	2084	0.000479846449136276
Co_P_Anthi	Coleoptera	Anthicidae	3000	0.000333333333333333
Co_P_Anthr	Coleoptera	Anthribidae	3900	0.000256410256410256
Co_P_Artem	Coleoptera_	Artematopodidae	45	0.0222222222222222
Co_P_Attel	Coleoptera	Attelabidae	2500	0.0004
Co_P_Belid	Coleoptera	Belidae	375	0.00266666666666667
Co_P_Biphy	Coleoptera	Biphyllidae	200	0.005
Co_P_Borid	Coleoptera	Boridae	4	0.25
Co_P_Bostr	Coleoptera	Bostrichidae	570	0.00175438596491228
Co_P_Bothr	Coleoptera	Bothrideridae	400	0.0025
Co_P_Brach	Coleoptera	Brachyceridae	385	0.0025974025974026
Co_P_Brent	Coleoptera	Brentidae	4000	0.00025
Co_P_Bupre	Coleoptera	Buprestidae	14700	0.0000680272108843537
Co_P_Byrrh	Coleoptera	Byrrhidae	430	0.00232558139534884
Co_P_Bytur	Coleoptera	Byturidae	24	0.0416666666666667
Co_P_Calli	Coleoptera	Callirhipidae	150	0.00666666666666667

Co_P_Canth	Coleoptera	Cantharidae	5100	0.000196078431372549
Co_P_Cepha	Coleoptera	Cephaloidae	19	0.0526315789473684
Co_P_Ceram	Coleoptera	Cerambycidae	30079	0.0000332457860966123
Co_P_Cerat	Coleoptera	Ceratocanthidae	120	0.00833333333333333
Co_P_Ceryl	Coleoptera	Cerylonidae	450	0.00222222222222222
Co_P_Chelo	Coleoptera	Chelonariidae	250	0.004
Co_P_Chrys	Coleoptera	Chrysomelidae	32500	0.0000307692307692308
Co_P_Ciida	Coleoptera	Ciidae	650	0.00153846153846154
Co_P_Clamb	Coleoptera	Clambidae	170	0.00588235294117647
Co_P_Cleri	Coleoptera	Cleridae	3400	0.000294117647058824
Co_P_Cocci	Coleoptera	Coccinellidae	6000	0.000166666666666667
Co_P_Coryl	Coleoptera	Corylophidae	200	0.005
Co_P_Crypt	Coleoptera	Cryptophagidae	600	0.00166666666666667
Co_P_Cucuj	Coleoptera	Cucujidae	44	0.0227272727272727
Co_P_Curcu	Coleoptera	Curculionidae	50615	0.0000197569890348711
Co_P_Dasci	Coleoptera	Dascillidae	80	0.0125
Co_P_Derme	Coleoptera	Dermestidae	1200	0.000833333333333333
Co_P_Derod	Coleoptera	Derodontidae	30	0.0333333333333333
Co_P_Disco	Coleoptera	Discolomatidae	400	0.0025
Co_P_Drili	Coleoptera	Drilidae	120	0.00833333333333333
Co_P_Dryop	Coleoptera	Dryopidae	300	0.00333333333333333
Co_P_Elate	Coleoptera	Elateridae	10000	0.0001
Co_P_Elmid	Coleoptera	Elmidae	1500	0.000666666666666667
Co_P_Endom	Coleoptera	Endomychidae	1800	0.000555555555555556
Co_P_Epime	Coleoptera	Epimetopidae	27	0.037037037037037
Co_P_Eroty	Coleoptera	Erotylidae	2500	0.0004
Co_P_Eucin	Coleoptera	Eucinetidae	53	0.0188679245283019
Co_P_Eucne	Coleoptera	Eucnemidae	1500	0.000666666666666667
Co_P_Eulic	Coleoptera	Eulichadidae	30	0.0333333333333333
Co_P_Geori	Coleoptera	Georissidae	77	0.012987012987013
Co_P_Geotr	Coleoptera	Geotrupidae	920	0.00108695652173913
Co_P_Glaph	Coleoptera	Glaphyridae	204	0.00490196078431373
Co_P_Glare	Coleoptera	Glaresidae	57	0.0175438596491228
Co_P_Helop	Coleoptera	Helophoridae	183	0.00546448087431694
Co_P_Helot	Coleoptera	Helotidae	107	0.00934579439252336
Co_P_Heter	Coleoptera	Heteroceridae	300	0.00333333333333333
Co_P_Histe	Coleoptera	Histeridae	4300	0.000232558139534884
Co_P_Hybos	Coleoptera	Hybosoridae	572	0.00174825174825175
Co_P_Hydra	Coleoptera	Hydraenidae	1600	0.000625
Co_P_Hydroc	Coleoptera	Hydrochidae	164	0.00609756097560976
Co_P_Hydrop	Coleoptera	Hydrophilidae	3400	0.000294117647058824
Co_P_Ithyc	Coleoptera	Ithyceridae	6	0.166666666666667
Co_P_Kater	Coleoptera	Kateretidae	95	0.0105263157894737
Co_P_Laemo	Coleoptera	Laemophloeidae	430	0.00232558139534884
Co_P_Lampy	Coleoptera	Lampyridae	2200	0.000454545454545455
Co_P_Langu	Coleoptera	Languriidae	1000	0.001
Co_P_Latri	Coleoptera	Latridiidae	1000	0.001
Co_P_Leiod	Coleoptera	Leiodidae	3700	0.00027027027027027
Co_P_Limni	Coleoptera	Limnichidae	390	0.00256410256410256

Co_P_Lucan	Coleoptera	Lucanidae	1489	0.000671591672263264
Co_P_Lutro	Coleoptera	Lutrochidae	11	0.0909090909090909
Co_P_Lycid	Coleoptera	Lycidae	4600	0.000217391304347826
Co_P_Lymex	Coleoptera	Lymexylidae	70	0.0142857142857143
Co_P_Mauro	Coleoptera	Mauroniscidae	26	0.0384615384615385
Co_P_Melan	Coleoptera	Melandryidae	420	0.00238095238095238
Co_P_Meloi	Coleoptera	Meloidae	3000	0.000333333333333333
Co_P_Melyr	Coleoptera	Melyridae	6000	0.000166666666666667
Co_P_Monot	Coleoptera	Monotomidae	250	0.004
Co_P_Morde	Coleoptera	Mordellidae	1500	0.000666666666666667
Co_P_Mycet	Coleoptera	Mycetophagidae	130	0.00769230769230769
Co_P_Nemon	Coleoptera	Nemonychidae	70	0.0142857142857143
Co_P_Nitid	Coleoptera	Nitidulidae	4500	0.000222222222222222
Co_P_Nosod	Coleoptera	Nosodendridae	50	0.02
Co_P_Ochod	Coleoptera	Ochodaeidae	110	0.00909090909090909
Co_P_Oedem	Coleoptera	Oedemeridae	500	0.002
Co_P_Omali	Coleoptera	Omalisidae	8	0.125
Co_P_Ometh	Coleoptera	Omethidae	33	0.0303030303030303
Co_P_Orsod	Coleoptera	Orsodacnidae	40	0.025
Co_P_Passal	Coleoptera	Passalidae	800	0.00125
Co_P_Passan	Coleoptera	Passandridae	109	0.00917431192660551
Co_P_Perim	Coleoptera	Perimylopidae	19	0.0526315789473684
Co_P_Phala	Coleoptera	Phalacridae	640	0.0015625
Co_P_Pheng	Coleoptera	Phengodidae	250	0.004
Co_P_Phloe	Coleoptera	Phloeostichidae	14	0.0714285714285714
Co_P_Phloi	Coleoptera	Phloiophilidae	1	1
Co_P_Pleoc	Coleoptera	Pleocomidae	50	0.02
Co_P_Prion	Coleoptera	Prionoceridae	160	0.00625
Co_P_Propa	Coleoptera	Propalticidae	30	0.0333333333333333
Co_P_Prost	Coleoptera	Prostomidae	30	0.0333333333333333
Co_P_Proto	Coleoptera	Protocucujidae	7	0.142857142857143
Co_P_Pseph	Coleoptera	Psephenidae	290	0.00344827586206897
Co_P_Ptili	Coleoptera	Ptiliidae	650	0.00153846153846154
Co_P_Ptilo	Coleoptera	Ptilodactylidae	500	0.002
Co_P_Ptini	Coleoptera	Ptinidae	500	0.002
Co_P_Pyroc	Coleoptera	Pyrochroidae	167	0.00598802395209581
Co_P_Pythi	Coleoptera	Pythidae	23	0.0434782608695652
Co_P_Rhipi	Coleoptera	Rhipiceridae	70	0.0142857142857143
Co_P_Ripip	Coleoptera	Ripiphoridae	400	0.0025
Co_P_Salpi	Coleoptera	Salpingidae	300	0.00333333333333333
Co_P_Scara	Coleoptera	Scarabaeidae	27000	0.000037037037037037
Co_P_Scirt	Coleoptera	Scirtidae	800	0.00125
Co_P_Scrap	Coleoptera	Scraptiidae	500	0.002
Co_P_Scydm	Coleoptera	Scydmaenidae	4586	0.000218054949847362
Co_P_Silph	Coleoptera	Silphidae	200	0.005
Co_P_Silva	Coleoptera	Silvanidae	500	0.002
Co_P_Sperc	Coleoptera	Spercheidae	19	0.0526315789473684
Co_P_Sphae	Coleoptera	Sphaeritidae	5	0.2
Co_P_Sphin	Coleoptera	Sphindidae	59	0.0169491525423729

Co_P_Staph	Coleoptera	Staphylinidae	56000	0.0000178571428571429
Co_P_Synch	Coleoptera	Synchroidae	8	0.125
Co_P_Teneb	Coleoptera	Tenebrionidae	20000	0.00005
Co_P_Tetra	Coleoptera	Tetratomidae	150	0.00666666666666667
Co_P_Thros	Coleoptera	Throscidae	150	0.00666666666666667
Co_P_Trict	Coleoptera	Trictenotomidae	13	0.0769230769230769
Co_P_Trogo	Coleoptera	Trogossitidae	600	0.00166666666666667
Co_P_Zophe	Coleoptera	Zopheridae	1700	0.000588235294117647
Coll_Bourl	Collembola	Bourletiellidae	245	0.00408163265306122
Coll_Brach	Collembola	Brachystomellidae	129	0.00775193798449612
Coll_Dicyr	Collembola	Dicyrtomidae	200	0.005
Coll_Entom	Collembola	Entomobryidae	1678	0.000595947556615018
Coll_Hypog	Collembola	Hypogastruridae	682	0.00146627565982405
Coll_Isoto	Collembola	Isotomidae	1346	0.000742942050520059
Coll_Katia	Collembola	Katiannidae	205	0.0048780487804878
Coll_Neanu	Collembola	Neanuridae	1417	0.000705716302046577
Coll_Neeli	Collembola	Neelidae	33	0.0303030303030303
Coll_Odont	Collembola	Odontellidae	131	0.00763358778625954
Coll_Oncop	Collembola	Oncopoduridae	52	0.0192307692307692
Coll_Onych	Collembola	Onychiuridae	567	0.0017636684303351
Coll_Paron	Collembola	Paronellidae	511	0.00195694716242661
Coll_Podur	Collembola	Poduridae	1	1
Coll_Smint	Collembola	Sminthuridae	245	0.00408163265306122
Coll_Tomoc	Collembola	Tomoceridae	149	0.00671140939597315
Coll_Tullb	Collembola	Tullbergiidae	215	0.00465116279069767
Der_Anisol	Dermaptera	Anisolabididae	38	0.0263157894736842
Der_Apachy	Dermaptera	Apachyidae	15	0.0666666666666667
Der_Chelis	Dermaptera	Chelisochidae	95	0.0105263157894737
Der_Forfic	Dermaptera	Forficulidae	485	0.00206185567010309
Der_Labidu	Dermaptera	Labiduridae	64	0.015625
Der_Labiid	Dermaptera	Labiidae	495	0.00202020202020202
Der_Pygidi	Dermaptera	Pygidicranidae	181	0.00552486187845304
Dipl_Campo	Diplura	Campodeidae	448	0.00223214285714286
Dipl_Japyg	Diplura	Japygidae	476	0.00210084033613445
Dipl_Para	Diplura	Parajapygidae	62	0.0161290322580645
Dipl_Proj	Diplura	Projapygoidea	52	0.0192307692307692
Di_Acartop	Diptera	Acartophthalmidae	6	0.166666666666667
Di_Acrocer	Diptera	Acroceridae	400	0.0025
Di_Agromyz	Diptera	Agromyzidae	3017	0.000331455087835598
Di_Anisop	Diptera	Anisopodidae	196	0.00510204081632653
Di_Anthomyi	Diptera	Anthomyiidae	1941	0.000515198351365276
Di_Anthomyz	Diptera	Anthomyzidae	100	0.01
Di_Apiocer	Diptera	Apioceridae	143	0.00699300699300699
Di_Apsiloc	Diptera	Apsilocephalidae	7	0.142857142857143
Di_Asilida	Diptera	Asilidae	7531	0.000132784490771478
Di_Asteiid	Diptera	Asteiidae	138	0.0072463768115942
Di_Atelest	Diptera	Atelestidae	22	0.0454545454545455
Di_Atheric	Diptera	Athericidae	133	0.0075187969924812
Di_Aulacig	Diptera	Aulacigastridae	19	0.0526315789473684

Di_Austral	Diptera	Australimyzidae	9	0.111111111111111
Di_Austrol	Diptera	Austroleptidae	8	0.125
Di_Axymyii	Diptera	Axymyiidae	8	0.125
Di_Bibioni	Diptera	Bibionidae	1102	0.000907441016333938
Di_Blephar	Diptera	Blephariceridae	331	0.00302114803625378
Di_Bombyli	Diptera	Bombyliidae	5382	0.000185804533630621
Di_Braulid	Diptera	Braulidae	7	0.142857142857143
Di_Calliph	Diptera	Calliphoridae	1525	0.000655737704918033
Di_Canthy	Diptera	Canthyloscelidae	14	0.0714285714285714
Di_Carnida	Diptera	Carnidae	92	0.0108695652173913
Di_Cecidom	Diptera	Cecidomyiidae	6296	0.000158831003811944
Di_Ceratop	Diptera	Ceratopogonidae	5902	0.000169434090138936
Di_Chaobor	Diptera	Chaoboridae	89	0.0112359550561798
Di_Chirono	Diptera	Chironomidae	7290	0.000137174211248285
Di_Chlorop	Diptera	Chloropidae	2885	0.000346620450606586
Di_Chyromy	Diptera	Chyromyidae	139	0.00719424460431655
Di_Clusiid	Diptera	Clusiidae	363	0.00275482093663912
Di_Coelopi	Diptera	Coelopidae	35	0.0285714285714286
Di_Conopid	Diptera	Conopidae	831	0.00120336943441637
Di_Corethr	Diptera	Corethrellidae	111	0.00900900900900901
Di_Culicid	Diptera	Culicidae	3725	0.000268456375838926
Di_Deutero	Diptera	Deuterophlebiidae	14	0.0714285714285714
Di_Diadoci	Diptera	Diadocidiidae	39	0.0256410256410256
Di_Diopsid	Diptera	Diopsidae	194	0.00515463917525773
Di_Dixidae	Diptera	Dixidae	197	0.0050761421319797
Di_Dolicho	Diptera	Dolichopodidae	7358	0.000135906496330525
Di_Drosoph	Diptera	Drosophilidae	4017	0.000248941996514812
Di_Dryomyz	Diptera	Dryomyzidae	30	0.0333333333333333
Di_Empidid	Diptera	Empididae	3142	0.000318268618714195
Di_Ephydri	Diptera	Ephydridae	1994	0.000501504513540622
Di_Fanniid	Diptera	Fanniidae	359	0.00278551532033426
Di_Ferguso	Diptera	Fergusoninidae	29	0.0344827586206897
Di_Glossin	Diptera	Glossinidae	25	0.04
Di_Helcomy	Diptera	Helcomyzidae	12	0.0833333333333333
Di_Helosci	Diptera	Helosciomyzidae	23	0.0434782608695652
Di_Hesperi	Diptera	Hesperinidae	10	0.1
Di_Heteroc	Diptera	Heterocheilidae	2	0.5
Di_Hilarim	Diptera	Hilarimorphidae	36	0.0277777777777778
Di_Hippobo	Diptera	Hippoboscidae	271	0.003690036900369
Di_Hybotid	Diptera	Hybotidae	2005	0.000498753117206983
Di_Keropla	Diptera	Keroplatidae	993	0.00100704934541793
Di_Lauxani	Diptera	Lauxaniidae	1900	0.000526315789473684
Di_Lonchae	Diptera	Lonchaeidae	504	0.00198412698412698
Di_Lonchop	Diptera	Lonchopteridae	65	0.0153846153846154
Di_Lygisto	Diptera	Lygistorrhinidae	44	0.0227272727272727
Di_Margin	Diptera	Marginidae	3	0.333333333333333
Di_Micrope	Diptera	Micropezidae	583	0.00171526586620926
Di_Milichi	Diptera	Milichiidae	288	0.00347222222222222
Di_Muscida	Diptera	Muscidae	5218	0.000191644308164048

Di_Mycetop	Diptera	Mycetophilidae	4525	0.000220994475138122
Di_Mydidae	Diptera	Mydidae	498	0.00200803212851406
Di_Mythico	Diptera	Mythicomyiidae	350	0.00285714285714286
Di_Nemestr	Diptera	Nemestrinidae	300	0.00333333333333333
Di_Neuroch	Diptera	Neurochaetidae	22	0.0454545454545455
Di_Nycteri	Diptera	Nycteribiidae	274	0.00364963503649635
Di_Odiniid	Diptera	Odiniidae	65	0.0153846153846154
Di_Oestrid	Diptera	Oestridae	176	0.00568181818181818
Di_Opomyzi	Diptera	Opomyzidae	61	0.0163934426229508
Di_Pachyne	Diptera	Pachyneuridae	8	0.125
Di_Pallopt	Diptera	Pallopteridae	71	0.0140845070422535
Di_Pelecor	Diptera	Pelecorhynchidae	49	0.0204081632653061
Di_Perisce	Diptera	Periscelididae	91	0.010989010989011
Di_Perisso	Diptera	Perissommatidae	9	0.111111111111111
Di_Phorida	Diptera	Phoridae	4200	0.000238095238095238
Di_Piophil	Diptera	Piophilidae	83	0.0120481927710843
Di_Pipuncu	Diptera	Pipunculidae	1428	0.000700280112044818
Di_Platype	Diptera	Platypezidae	277	0.0036101083032491
Di_Platyst	Diptera	Platystomatidae	1164	0.000859106529209622
Di_Pleciid	Diptera	Pleciidae	280	0.00357142857142857
Di_Psilida	Diptera	Psilidae	322	0.0031055900621118
Di_Psychod	Diptera	Psychodidae	3026	0.000330469266358229
Di_Ptychop	Diptera	Ptychopteridae	156	0.00641025641025641
Di_Pyrgoti	Diptera	Pyrgotidae	351	0.00284900284900285
Di_Rhagion	Diptera	Rhagionidae	756	0.00132275132275132
Di_Rhinoph	Diptera	Rhinophoridae	174	0.00574712643678161
Di_Richar	Diptera	Richardiidae	178	0.00561797752808989
Di_Sarcoph	Diptera	Sarcophagidae	3094	0.000323206205559147
Di_Scathop	Diptera	Scathophagidae	419	0.00238663484486874
Di_Scatops	Diptera	Scatopsidae	407	0.00245700245700246
Di_Scenopi	Diptera	Scenopinidae	420	0.00238095238095238
Di_Sciadoc	Diptera	Sciardoceridae	2	0.5
Di_Sciarid	Diptera	Sciaridae	2455	0.000407331975560081
Di_Sciomyz	Diptera	Sciomyzidae	618	0.00161812297734628
Di_Sepsida	Diptera	Sepsidae	345	0.00289855072463768
Di_Simulii	Diptera	Simuliidae	2121	0.000471475719000471
Di_Somatid	Diptera	Somatiidae	7	0.142857142857143
Di_Sphaero	Diptera	Sphaeroceridae	1571	0.00063653723742839
Di_Stratio	Diptera	Stratiomyidae	2690	0.000371747211895911
Di_Strebli	Diptera	Streblidae	237	0.00421940928270042
Di_Strongy	Diptera	Strongylophthalmyiidae	45	0.0222222222222222
Di_Synneur	Diptera	Synneuridae	3	0.333333333333333
Di_Syrphid	Diptera	Syrphidae	6107	0.000163746520386442
Di_Tabanid	Diptera	Tabanidae	4434	0.0002255299954894
Di_Tachini	Diptera	Tachinidae	9626	0.000103885310617079
Di_Tanyder	Diptera	Tanyderidae	55	0.0181818181818182
Di_Tephrit	Diptera	Tephritidae	4716	0.000212044105173876
Di_Thaumal	Diptera	Thaumaleidae	183	0.00546448087431694
Di_Therevi	Diptera	Therevidae	1143	0.000874890638670166

Di_Tipulid	Diptera	Tipulidae	15770	0.0000634115409004439
Di_Trichoc	Diptera	Trichoceridae	183	0.00546448087431694
Di_Ulidiid	Diptera	Ulidiidae	678	0.00147492625368732
Di_Vermile	Diptera	Vermileonidae	61	0.0163934426229508
Di_Xenaste	Diptera	Xenasteiidae	13	0.0769230769230769
Di_Xylomyi	Diptera	Xylomyidae	138	0.0072463768115942
Di_Xylopha	Diptera	Xylophagidae	145	0.00689655172413793
Emb_Anisem	Embioptera	Anisembiidae	105	0.00952380952380952
Emb_Austra	Embioptera	Australembiidae	31	0.032258064516129
Emb_Clotho	Embioptera	Clothodidae	15	0.0666666666666667
Emb_Embiid	Embioptera	Embiidae	83	0.0120481927710843
Emb_Notoli	Embioptera	Notoligotomidae	3	0.333333333333333
Emb_Oligot	Embioptera	Oligotomidae	52	0.0192307692307692
Emb_Terate	Embioptera	Teratembiidae	48	0.0208333333333333
Ep_Acantha	Ephemeroptera	Acanthametropodidae	3	0.333333333333333
Ep_Ameleti	Ephemeroptera	Ameletidae	56	0.0178571428571429
Ep_Ameleto	Ephemeroptera	Ameletopsidae	6	0.166666666666667
Ep_Ametrop	Ephemeroptera	Ametropodidae	3	0.333333333333333
Ep_Baetida	Ephemeroptera	Baetidae	860	0.00116279069767442
Ep_Baetisc	Ephemeroptera	Baetiscidae	12	0.0833333333333333
Ep_Behning	Ephemeroptera	Behningiidae	7	0.142857142857143
Ep_Caenida	Ephemeroptera	Caenidae	211	0.004739336492891
Ep_Colobur	Ephemeroptera	Coloburiscidae	6	0.166666666666667
Ep_Diptero	Ephemeroptera	Dipteromimidae	2	0.5
Ep_Ephemerel	Ephemeroptera	Ephemerellidae	160	0.00625
Ep_Ephemeri	Ephemeroptera	Ephemeridae	91	0.010989010989011
Ep_Euthypl	Ephemeroptera	Euthyplociidae	19	0.0526315789473684
Ep_Heptage	Ephemeroptera	Heptageniidae	529	0.00189035916824197
Ep_Ichthyb	Ephemeroptera	Ichthybotidae	2	0.5
Ep_Isonych	Ephemeroptera	Isonychiidae	30	0.0333333333333333
Ep_Leptohy	Ephemeroptera	Leptohyphidae	157	0.00636942675159236
Ep_Leptoph	Ephemeroptera	Leptophlebiidae	623	0.00160513643659711
Ep_Metreto	Ephemeroptera	Metretopodidae	13	0.0769230769230769
Ep_Neoephe	Ephemeroptera	Neoephemeridae	7	0.142857142857143
Ep_Nesamel	Ephemeroptera	Nesameletidae	11	0.0909090909090909
Ep_Oligone	Ephemeroptera	Oligoneuriidae	54	0.0185185185185185
Ep_Oniscig	Ephemeroptera	Oniscigastridae	8	0.125
Ep_Palinge	Ephemeroptera	Palingeniidae	32	0.03125
Ep_Polymit	Ephemeroptera	Polymitarcyidae	84	0.0119047619047619
Ep_Potaman	Ephemeroptera	Potamanthidae	23	0.0434782608695652
Ep_Prosopi	Ephemeroptera	Prosopistomatidae	19	0.0526315789473684
Ep_Rallide	Ephemeroptera	Rallidentidae	1	1
Ep_Siphlae	Ephemeroptera	Siphlaenigmatidae	1	1
Ep_Siphlon	Ephemeroptera	Siphlonuridae	49	0.0204081632653061
Ep_Telogan	Ephemeroptera	Teloganodidae	13	0.0769230769230769
Ep_Tricory	Ephemeroptera	Tricorythidae	34	0.0294117647058824
Grylloblat	Grylloblattidae	Grylloblattidae	27	0.037037037037037
He_Acantho	Hemiptera	Acanthosomatidae	200	0.005
He_Achilid	Hemiptera	Achilidae	503	0.00198807157057654

He_Achilix	Hemiptera	Achilixiidae	24	0.0416666666666667
He_Aetalio	Hemiptera	Aetalionidae	42	0.0238095238095238
He_Aleyrod	Hemiptera	Aleyrodoidea	1560	0.000641025641025641
He_Alydida	Hemiptera	Alydidae	250	0.004
He_Anthoco	Hemiptera	Anthocoridae	600	0.00166666666666667
He_Apheloc	Hemiptera	Aphelocheiridae	400	0.0025
He_Aphidoi	Hemiptera	Aphidoidea	4300	0.000232558139534884
He_Aphroph	Hemiptera	Aphrophoridae	820	0.00121951219512195
He_Aradida	Hemiptera	Aradidae	2000	0.0005
He_Belosto	Hemiptera	Belostomatidae	150	0.00666666666666667
He_Berytid	Hemiptera	Berytidae	100	0.01
He_Calisce	Hemiptera	Caliscelidae	202	0.00495049504950495
He_Canopid	Hemiptera	Canopidae	8	0.125
He_Cercopi	Hemiptera	Cercopidae	1400	0.000714285714285714
He_Cicadel	Hemiptera	Cicadellidae	20000	0.00005
He_Cicadid	Hemiptera	Cicadidae	1300	0.000769230769230769
He_Cimicid	Hemiptera	Cimicidae	100	0.01
He_Cixiida	Hemiptera	Cixiidae	2223	0.000449842555105713
He_Clastop	Hemiptera	Clastopteridae	80	0.0125
He_Coccoid	Hemiptera	Coccoidea	8000	0.000125
He_Colobat	Hemiptera	Colobathristidae	90	0.0111111111111111
He_Coreida	Hemiptera	Coreidae	1900	0.000526315789473684
He_Corixid	Hemiptera	Corixidae	600	0.00166666666666667
He_Cydnida	Hemiptera	Cydnidae	617	0.00162074554294976
He_Delphac	Hemiptera	Delphacidae	2029	0.000492853622474125
He_Derbida	Hemiptera	Derbidae	1700	0.000588235294117647
He_Dictyop	Hemiptera	Dictyopharidae	731	0.00136798905608755
He_Dinidor	Hemiptera	Dinidoridae	90	0.0111111111111111
He_Dipsoco	Hemiptera	Dipsocoridae	30	0.0333333333333333
He_Epipygi	Hemiptera	Epipygidae	27	0.037037037037037
He_Enicoce	Hemiptera	Enicocephalidae	400	0.0025
He_Eurybra	Hemiptera	Eurybrachyidae	189	0.00529100529100529
He_Flatida	Hemiptera	Flatidae	1446	0.000691562932226833
He_Fulgori	Hemiptera	Fulgoridae	687	0.00145560407569141
He_Gelasto	Hemiptera	Gelastocoridae	100	0.01
He_Gerrida	Hemiptera	Gerridae	620	0.00161290322580645
He_Hebrida	Hemiptera	Hebridae	150	0.00666666666666667
He_Hermato	Hemiptera	Hermatobatidae	8	0.125
He_Hydrome	Hemiptera	Hydrometridae	110	0.00909090909090909
He_Hyoceph	Hemiptera	Hyocephalidae	3	0.333333333333333
He_Idiosto	Hemiptera	Idiostolidae	4	0.25
He_Issidae	Hemiptera	Issidae	924	0.00108225108225108
He_Joppeic	Hemiptera	Joppeicidae	1	1
He_Largida	Hemiptera	Largidae	120	0.00833333333333333
He_Leptopo	Hemiptera	Leptopodidae	40	0.025
He_Lestoni	Hemiptera	Lestoniidae	2	0.5
He_Lophopi	Hemiptera	Lophopidae	138	0.0072463768115942
He_Lycetoc	Hemiptera	Lyctocoridae	27	0.037037037037037
He_Lygaeio	Hemiptera	Lygaeidae	4400	0.000227272727272727

He_Machaer	Hemiptera	Machaerotidae	110	0.00909090909090909
He_Macrove	Hemiptera	Macroveliidae	3	0.333333333333333
He_Malcida	Hemiptera	Malcidae	20	0.05
He_Meenopl	Hemiptera	Meenoplidae	158	0.00632911392405063
He_Membrac	Hemiptera	Membracidae	3450	0.000289855072463768
He_Mesovel	Hemiptera	Mesoveliidae	35	0.0285714285714286
He_Microph	Hemiptera	Microphysidae	30	0.0333333333333333
He_Miridae	Hemiptera	Miridae	10000	0.0001
He_Myerslo	Hemiptera	Myerslopiidae	20	0.05
He_Nabidae	Hemiptera	Nabidae	400	0.0025
He_Naucori	Hemiptera	Naucoridae	500	0.002
He_Nepidae	Hemiptera	Nepidae	225	0.00444444444444444
He_Nogodin	Hemiptera	Nogodinidae	286	0.0034965034965035
He_Notonec	Hemiptera	Notonectidae	350	0.00285714285714286
He_Ochteri	Hemiptera	Ochteridae	50	0.02
He_Paraphy	Hemiptera	Paraphrynoveliidae	2	0.5
He_Pelorid	Hemiptera	Peloridiidae	12	0.0833333333333333
He_Pentato	Hemiptera	Pentatomidae	4500	0.000222222222222222
He_Phloeid	Hemiptera	Phloeidae	3	0.333333333333333
He_Phyllox	Hemiptera	Phylloxeroidea	75	0.0133333333333333
He_Phymati	Hemiptera	Phymatidae	280	0.00357142857142857
He_Piesmat	Hemiptera	Piesmatidae	40	0.025
He_Platasp	Hemiptera	Plataspidae	500	0.002
He_Pleidae	Hemiptera	Pleidae	40	0.025
He_Plokiop	Hemiptera	Plokiophilidae	6	0.166666666666667
He_Psylloi	Hemiptera	Psylloidea	2500	0.0004
He_Pyrrhoc	Hemiptera	Pyrrhocoridae	225	0.00444444444444444
He_Reduvii	Hemiptera	Reduviidae	6420	0.000155763239875389
He_Rhopali	Hemiptera	Rhopalidae	200	0.005
He_Ricanni	Hemiptera	Ricaniidae	417	0.00239808153477218
He_Saldida	Hemiptera	Saldidae	265	0.00377358490566038
He_Schizop	Hemiptera	Schizopteridae	120	0.00833333333333333
He_Scutell	Hemiptera	Scutelleridae	500	0.002
He_Stenoce	Hemiptera	Stenocephalidae	30	0.0333333333333333
He_Termita	Hemiptera	Termitaphididae	9	0.111111111111111
He_Tessara	Hemiptera	Tessaratomidae	250	0.004
He_Tettigo	Hemiptera	Tettigometridae	73	0.0136986301369863
He_Thaumas	Hemiptera	Thaumastocoridae	19	0.0526315789473684
He_Tingida	Hemiptera	Tingidae	2000	0.0005
He_Tropidu	Hemiptera	Tropiduchidae	575	0.00173913043478261
He_Veliida	Hemiptera	Veliidae	720	0.00138888888888889
He_Velocip	Hemiptera	Velocipedidae	31	0.032258064516129
Hy_Agaonid	Hymenoptera	Agaonidae	757	0.00132100396301189
Hy_Ampulic	Hymenoptera	Ampulicidae	200	0.005
Hy_Anaxeli	Hymenoptera	Anaxyelidae	1	1
Hy_Andreni	Hymenoptera	Andrenidae	2938	0.000340367597004765
Hy_Aphelin	Hymenoptera	Aphelinidae	1168	0.000856164383561644
Hy_Apidae	Hymenoptera	Apidae	5751	0.000173882802990784
Hy_Argidae	Hymenoptera	Argidae	800	0.00125

Hy_Aulacid	Hymenoptera	Aulacidae	200	0.005
Hy_Bethyli	Hymenoptera	Bethylidae	2000	0.0005
Hy_Blastic	Hymenoptera	Blasticotomidae	10	0.1
Hy_Braconi	Hymenoptera	Braconidae	20000	0.00005
Hy_Bradyno	Hymenoptera	Bradynobaenidae	200	0.005
Hy_Cephida	Hymenoptera	Cephidae	80	0.0125
Hy_Ceraphr	Hymenoptera	Ceraphronidae	350	0.00285714285714286
Hy_Chalcid	Hymenoptera	Chalcididae	1464	0.000683060109289617
Hy_Chrysid	Hymenoptera	Chrysididae	3000	0.000333333333333333
Hy_Cimbici	Hymenoptera	Cimbicidae	130	0.00769230769230769
Hy_Colleti	Hymenoptera	Colletidae	2545	0.000392927308447937
Hy_Crabron	Hymenoptera	Crabronidae	8774	0.000113973102347846
Hy_Cynipid	Hymenoptera	Cynipidae	1000	0.001
Hy_Diaprii	Hymenoptera	Diapriidae	2300	0.000434782608695652
Hy_Diprion	Hymenoptera	Diprionidae	90	0.0111111111111111
Hy_Encyrti	Hymenoptera	Encyrtidae	3735	0.000267737617135208
Hy_Euchari	Hymenoptera	Eucharitidae	423	0.00236406619385343
Hy_Eulophi	Hymenoptera	Eulophidae	4472	0.000223613595706619
Hy_Eupelmi	Hymenoptera	Eupelmidae	907	0.00110253583241455
Hy_Eurytom	Hymenoptera	Eurytomidae	1424	0.000702247191011236
Hy_Evaniid	Hymenoptera	Evaniidae	500	0.002
Hy_Figitid	Hymenoptera	Figitidae	1500	0.000666666666666667
Hy_Formici	Hymenoptera	Formicidae	10000	0.0001
Hy_Gasteru	Hymenoptera	Gasteruptiidae	420	0.00238095238095238
Hy_Halicti	Hymenoptera	Halictidae	4338	0.000230520977408944
Hy_Helorid	Hymenoptera	Heloridae	7	0.142857142857143
Hy_Ibaliid	Hymenoptera	Ibaliidae	50	0.02
Hy_Ichneum	Hymenoptera	Ichneumonidae	22000	0.0000454545454545455
Hy_Liopter	Hymenoptera	Liopteridae	50	0.02
Hy_Maaming	Hymenoptera	Maamingidae	2	0.5
Hy_Megachi	Hymenoptera	Megachilidae	4120	0.000242718446601942
Hy_Megalod	Hymenoptera	Megalodontesidae	40	0.025
Hy_Megalyr	Hymenoptera	Megalyridae	50	0.02
Hy_Megaspi	Hymenoptera	Megaspilidae	450	0.00222222222222222
Hy_Melitti	Hymenoptera	Melittidae	191	0.00523560209424084
Hy_Monomac	Hymenoptera	Monomachidae	20	0.05
Hy_Mutilid	Hymenoptera	Mutillidae	5000	0.0002
Hy_Mymarid	Hymenoptera	Mymaridae	1424	0.000702247191011236
Hy_Mymaron	Hymenoptera	Mymarommatidae	9	0.111111111111111
Hy_Orussid	Hymenoptera	Orussidae	75	0.0133333333333333
Hy_Pamphil	Hymenoptera	Pamphiliidae	250	0.004
Hy_Pelecin	Hymenoptera	Pelecinidae	3	0.333333333333333
Hy_Pergida	Hymenoptera	Pergidae	500	0.002
Hy_Perilam	Hymenoptera	Perilampidae	277	0.0036101083032491
Hy_Platyga	Hymenoptera	Platygastridae	1100	0.000909090909090909
Hy_Plumari	Hymenoptera	Plumariidae	20	0.05
Hy_Pompili	Hymenoptera	Pompilidae	4000	0.00025
Hy_Proctot	Hymenoptera	Proctotrupidae	310	0.00322580645161290
Hy_Pteroma	Hymenoptera	Pteromalidae	3506	0.000285225328009127

Hy_Roprion	Hymenoptera	Roproniidae	18	0.0555555555555556
Hy_Rotoiti	Hymenoptera	Rotoitidae	2	0.5
Hy_Sapygid	Hymenoptera	Sapygidae	80	0.0125
Hy_Scelion	Hymenoptera	Scelionidae	3000	0.000333333333333333
Hy_Scoleby	Hymenoptera	Scolebythidae	3	0.333333333333333
Hy_Scoliid	Hymenoptera	Scoliidae	300	0.00333333333333333
Hy_Siricid	Hymenoptera	Siricidae	95	0.0105263157894737
Hy_Sierolo	Hymenoptera	Sierolomorphidae	10	0.1
Hy_Sphecid	Hymenoptera	Sphecidae	724	0.00138121546961326
Hy_Stenotr	Hymenoptera	Stenotritidae	21	0.0476190476190476
Hy_Stephan	Hymenoptera	Stephanidae	200	0.005
Hy_Tenthre	Hymenoptera	Tenthredinidae	4000	0.00025
Hy_Tetraca	Hymenoptera	Tetracampidae	50	0.02
Hy_Tiphiid	Hymenoptera	Tiphiidae	1500	0.000666666666666667
Hy_Torymid	Hymenoptera	Torymidae	986	0.00101419878296146
Hy_Trichog	Hymenoptera	Trichogrammatidae	839	0.00119189511323004
Hy_Trigona	Hymenoptera	Trigonalidae	100	0.01
Hy_Vanhorn	Hymenoptera	Vanhorniidae	5	0.2
Hy_Vespida	Hymenoptera	Vespidae	4000	0.00025
Hy_Xiphydr	Hymenoptera	Xiphydriidae	100	0.01
Hy_Xyelida	Hymenoptera	Xyelidae	50	0.02
Is_Hodoter	Isoptera	Hodotermitidae	15	0.0666666666666667
Is_Kaloter	Isoptera	Kalotermitidae	420	0.00238095238095238
Is_Mastote	Isoptera	Mastotermitidae	1	1
Is_Rhinote	Isoptera	Rhinotermitidae	280	0.00357142857142857
Is_Serrite	Isoptera	Serritermitidae	6	0.166666666666667
Is_Termiti	Isoptera	Termitidae	1915	0.000522193211488251
Is_Termops	Isoptera	Termopsidae	21	0.0476190476190476
L_Acanthop	Lepidoptera	Acanthopteroctetidae	5	0.2
L_Acroloph	Lepidoptera	Acrolophidae	300	0.00333333333333333
L_Adelidae	Lepidoptera	Adelidae	294	0.00340136054421769
L_Agathiph	Lepidoptera	Agathiphagidae	2	0.5
L_Agonoxen	Lepidoptera	Agonoxenidae	4	0.25
L_Aididae	Lepidoptera	Aididae	6	0.166666666666667
L_Alucitid	Lepidoptera	Alucitidae	216	0.00462962962962963
L_Amphisba	Lepidoptera	Amphisbatidae	21	0.0476190476190476
L_Andesian	Lepidoptera	Andesianidae	3	0.333333333333333
L_Anomoeti	Lepidoptera	Anomoeotidae	40	0.025
L_Anthelid	Lepidoptera	Anthelidae	94	0.0106382978723404
L_Apatelod	Lepidoptera	Apatelodidae	145	0.00689655172413793
L_Arctiida	Lepidoptera	Arctiidae	6000	0.000166666666666667
L_Argyrest	Lepidoptera	Argyresthiidae	157	0.00636942675159236
L_Arrhenop	Lepidoptera	Arrhenophanidae	26	0.0384615384615385
L_Autostic	Lepidoptera	Autostichidae	585	0.00170940170940171
L_Batrache	Lepidoptera	Batrachedridae	99	0.0101010101010101
L_Bedellid	Lepidoptera	Bedelliidae	16	0.0625
L_Blastoba	Lepidoptera	Blastobasidae	377	0.0026525198938992
L_Bombycid	Lepidoptera	Bombycidae	185	0.00540540540540541
L_Brachodi	Lepidoptera	Brachodidae	137	0.0072992700729927

L_Brahmaei	Lepidoptera	Brahmaeidae	44	0.0227272727272727
L_Bucculat	Lepidoptera	Bucculatricidae	297	0.00336700336700337
L_Callidul	Lepidoptera	Callidulidae	49	0.0204081632653061
L_Carposin	Lepidoptera	Carposinidae	283	0.00353356890459364
L_Carthaei	Lepidoptera	Carthaeidae	1	1
L_Castniid	Lepidoptera	Castniidae	113	0.00884955752212389
L_Choreuti	Lepidoptera	Choreutidae	406	0.00246305418719212
L_Cimeliid	Lepidoptera	Cimeliidae	6	0.166666666666667
L_Coleopho	Lepidoptera	Coleophoridae	1386	0.000721500721500722
L_Copromor	Lepidoptera	Copromorphidae	43	0.0232558139534884
L_Cosmopte	Lepidoptera	Cosmopterigidae	1792	0.000558035714285714
L_Cossidae	Lepidoptera	Cossidae	971	0.00102986611740474
L_Crambida	Lepidoptera	Crambidae	9655	0.000103573278094252
L_Crinopte	Lepidoptera	Crinopterygidae	1	1
L_Cyclotor	Lepidoptera	Cyclotornidae	5	0.2
L_Dalcerid	Lepidoptera	Dalceridae	80	0.0125
L_Deocloni	Lepidoptera	Deoclonidae	4	0.25
L_Deuterog	Lepidoptera	Deuterogoniidae	4	0.25
L_Douglasi	Lepidoptera	Douglasiidae	29	0.0344827586206897
L_Drepanid	Lepidoptera	Drepanidae	660	0.00151515151515152
L_Dudgeoni	Lepidoptera	Dudgeoneidae	57	0.0175438596491228
L_Elachist	Lepidoptera	Elachistidae	3197	0.000312793243665937
L_Endromid	Lepidoptera	Endromidae	56	0.0178571428571429
L_Epicopei	Lepidoptera	Epicopeiidae	20	0.05
L_Epipyrop	Lepidoptera	Epipyropidae	32	0.03125
L_Ericotti	Lepidoptera	Eriocottidae	80	0.0125
L_Eriocran	Lepidoptera	Eriocraniidae	28	0.0357142857142857
L_Eupterot	Lepidoptera	Eupterotidae	339	0.00294985250737463
L_Gelechid	Lepidoptera	Gelechiidae	4700	0.000212765957446809
L_Geometri	Lepidoptera	Geometridae	23002	0.0000434744804799583
L_Glyphido	Lepidoptera	Glyphidoceridae	49	0.0204081632653061
L_Glyphipt	Lepidoptera	Glyphipterigidae	535	0.00186915887850467
L_Gracilla	Lepidoptera	Gracillariidae	1864	0.000536480686695279
L_Hedylida	Lepidoptera	Hedylidae	36	0.0277777777777778
L_Heliozel	Lepidoptera	Heliozelidae	123	0.00813008130081301
L_Hepialid	Lepidoptera	Hepialidae	604	0.00165562913907285
L_Hesperii	Lepidoptera	Hesperiidae	4113	0.000243131534159981
L_Heteroba	Lepidoptera	Heterobathmiidae	3	0.333333333333333
L_Heterogy	Lepidoptera	Heterogynidae	10	0.1
L_Himantop	Lepidoptera	Himantopteridae	40	0.025
L_Hyblaeid	Lepidoptera	Hyblaeidae	18	0.0555555555555556
L_Immidae	Lepidoptera	Immidae	245	0.00408163265306122
L_Incurvar	Lepidoptera	Incurvariidae	50	0.02
L_Lacturid	Lepidoptera	Lacturidae	120	0.00833333333333333
L_Lasiocam	Lepidoptera	Lasiocampidae	1952	0.000512295081967213
L_Lecithoc	Lepidoptera	Lecithoceridae	1200	0.000833333333333333
L_Lemoniid	Lepidoptera	Lemoniidae	21	0.0476190476190476
L_Limacodi	Lepidoptera	Limacodidae	1672	0.000598086124401914
L_Lycaenid	Lepidoptera	Lycaenidae	5201	0.000192270717169775

L_Lymantri	Lepidoptera	Lymantriidae	2500	0.0004
L_Lyoneti	Lepidoptera	Lyonetiidae	204	0.00490196078431373
L_Megalopy	Lepidoptera	Megalopygidae	232	0.00431034482758621
L_Micropte	Lepidoptera	Micropterigidae	154	0.00649350649350649
L_Mimallon	Lepidoptera	Mimallonidae	194	0.00515463917525773
L_Mirinida	Lepidoptera	Mirinidae	3	0.333333333333333
L_Mnesacha	Lepidoptera	Mnesarchaeidae	7	0.142857142857143
L_Momphida	Lepidoptera	Momphidae	115	0.00869565217391304
L_Neopseus	Lepidoptera	Neopseustidae	14	0.0714285714285714
L_Nepticul	Lepidoptera	Nepticulidae	806	0.00124069478908189
L_Noctuida	Lepidoptera	Noctuidae	30579	0.0000327021812354884
L_Notodont	Lepidoptera	Notodontidae	3800	0.000263157894736842
L_Nymphali	Lepidoptera	Nymphalidae	6131	0.000163105529277443
L_Oecophor	Lepidoptera	Oecophoridae	3304	0.000302663438256659
L_Opostegi	Lepidoptera	Opostegidae	192	0.00520833333333333
L_Palaepha	Lepidoptera	Palaephatidae	57	0.0175438596491228
L_Papilion	Lepidoptera	Papilionidae	566	0.00176678445229682
L_Peleopod	Lepidoptera	Peleopodidae	28	0.0357142857142857
L_Pieridae	Lepidoptera	Pieridae	1164	0.000859106529209622
L_Plutelli	Lepidoptera	Plutellidae	150	0.00666666666666667
L_Prodoxid	Lepidoptera	Prodoxidae	98	0.0102040816326531
L_Protothe	Lepidoptera	Prototheoridae	12	0.0833333333333333
L_Psychida	Lepidoptera	Psychidae	1324	0.000755287009063444
L_Pterolon	Lepidoptera	Pterolonchidae	8	0.125
L_Pteropho	Lepidoptera	Pterophoridae	1318	0.000758725341426404
L_Pyralida	Lepidoptera	Pyralidae	5921	0.000168890390136801
L_Riodinid	Lepidoptera	Riodinidae	1532	0.000652741514360313
L_Roeslers	Lepidoptera	Roeslerstammiidae	53	0.0188679245283019
L_Saturnii	Lepidoptera	Saturniidae	2349	0.00042571306939123
L_Sematuri	Lepidoptera	Sematuridae	40	0.025
L_Sesiidae	Lepidoptera	Sesiidae	1397	0.000715819613457409
L_Somabrac	Lepidoptera	Somabrachyidae	8	0.125
L_Sphingid	Lepidoptera	Sphingidae	1461	0.000684462696783025
L_Thyridid	Lepidoptera	Thyrididae	940	0.00106382978723404
L_Tineidae	Lepidoptera	Tineidae	2093	0.000477783086478739
L_Tineodid	Lepidoptera	Tineodidae	19	0.0526315789473684
L_Tischeri	Lepidoptera	Tischeriidae	110	0.00909090909090909
L_Tortrici	Lepidoptera	Tortricidae	10387	0.0000962741888899586
L_Uraniida	Lepidoptera	Uraniidae	686	0.00145772594752187
L_Urodidae	Lepidoptera	Urodidae	66	0.0151515151515152
L_Xyloryct	Lepidoptera	Xyloryctidae	524	0.00190839694656489
L_Yponomeu	Lepidoptera	Yponomeutidae	363	0.00275482093663912
L_Ypsolop	Lepidoptera	Ypsolophidae	163	0.00613496932515337
L_Zygaenid	Lepidoptera	Zygaenidae	1036	0.000965250965250965
Man_Acanth	Mantodea	Acanthopidae	80	0.0125
Man_Amorph	Mantodea	Amorphoscelidae	48	0.0208333333333333
Man_Chaete	Mantodea	Chaeteessidae	6	0.166666666666667
Man_Empusi	Mantodea	Empusidae	16	0.0625
Man_Eremia	Mantodea	Eremiaphilidae	68	0.0147058823529412

Man_Hymeno	Mantodea	Hymenopodidae	218	0.00458715596330275
Man_Iridop	Mantodea	Iridopterygidae	116	0.00862068965517241
Man_Liturg	Mantodea	Liturgusidae	73	0.0136986301369863
Man_Mantid	Mantodea	Mantidae	1114	0.000897666068222621
Man_Mantoi	Mantodea	Mantoididae	10	0.1
Man_Metall	Mantodea	Metallyticidae	5	0.2
Man_Paraox	Mantodea	Paraoxypilidae	30	0.0333333333333333
Man_Sibyll	Mantodea	Sibyllidae	16	0.0625
Man_Tarach	Mantodea	Tarachodidae	109	0.00917431192660551
Man_Thespi	Mantodea	Thespidae	202	0.00495049504950495
Man_Toxode	Mantodea	Toxoderidae	52	0.0192307692307692
Mantophasm	Mantophasmatodea	Mantophasmatodea	16	0.0625
Mec_Aptero	Mecoptera	Apteropanorpidae	1	1
Mec_Bittac	Mecoptera	Bittacidae	214	0.00467289719626168
Mec_Boreid	Mecoptera	Boreidae	38	0.0263157894736842
Mec_Choris	Mecoptera	Choristidae	12	0.0833333333333333
Mec_Merop	Mecoptera	Meropeidae	2	0.5
Mec_Nannoc	Mecoptera	Nannochoristidae	9	0.111111111111111
Mec_Panora	Mecoptera	Panorpidae	480	0.00208333333333333
Mec_Panorb	Mecoptera	Panorpodidae	19	0.0526315789473684
Meg_Coryda	Megaloptera	Corydalidae	200	0.005
Meg_Sialid	Megaloptera	Sialidae	70	0.0142857142857143
Neu_Ascala	Neuroptera	Ascalaphidae	430	0.00232558139534884
Neu_Beroth	Neuroptera	Berothidae	115	0.00869565217391304
Neu_Chryso	Neuroptera	Chrysopidae	1200	0.000833333333333333
Neu_Coniop	Neuroptera	Coniopterygidae	450	0.00222222222222222
Neu_Hemer	Neuroptera	Hemerobiidae	550	0.00181818181818182
Neu_Ithoni	Neuroptera	Ithonidae	53	0.0188679245283019
Neu_Mantis	Neuroptera	Mantispidae	400	0.0025
Neu_Myrmel	Neuroptera	Myrmeleontidae	2100	0.000476190476190476
Neu_Nemopt	Neuroptera	Nemopteridae	100	0.01
Neu_Nevror	Neuroptera	Nevrorthidae	12	0.0833333333333333
Neu_Nymphi	Neuroptera	Nymphidae	35	0.0285714285714286
Neu_Osmyli	Neuroptera	Osmylidae	160	0.00625
Neu_Polyst	Neuroptera	Polystoechotidae	4	0.25
Neu_Psych	Neuroptera	Psychopsidae	26	0.0384615384615385
Neu_Sisyri	Neuroptera	Sisyridae	50	0.02
Od_A_Aeshn	Odonata	Aeshnidae	428	0.00233644859813084
Od_A_Austr	Odonata	Austropetaliidae	11	0.0909090909090909
Od_A_Chlor	Odonata	Chlorogomphidae	45	0.0222222222222222
Od_A_Corda	Odonata	Cordulegastridae	51	0.0196078431372549
Od_A_Cordb	Odonata	Corduliidae	242	0.00413223140495868
Od_A_Gomph	Odonata	Gomphidae	945	0.00105820105820106
Od_A_Libel	Odonata	Libellulidae	970	0.00103092783505155
Od_A_Macro	Odonata	Macromiidae	123	0.00813008130081301
Od_A_Neope	Odonata	Neopetaliidae	1	1
Od_A_Petal	Odonata	Petaluridae	11	0.0909090909090909
Od_A_Synth	Odonata	Synthemistidae	43	0.0232558139534884
Od_AZ_Epio	Odonata	Epiophlebiidae	2	0.5

Od_Z_Amphy	Odonata	Amphyipterygidae	10	0.1
Od_Z_Calop	Odonata	Calopterygidae	172	0.00581395348837209
Od_Z_Chlor	Odonata	Chlorocyphidae	143	0.00699300699300699
Od_Z_Chori	Odonata	Chorismagrionidae	1	1
Od_Z_Coena	Odonata	Coenagrionidae	1104	0.000905797101449275
Od_Z_Dicte	Odonata	Dicteriadidae	2	0.5
Od_Z_Diphl	Odonata	Diphlebiidae	9	0.111111111111111
Od_Z_Eupha	Odonata	Euphaeidae	68	0.0147058823529412
Od_Z_Hemip	Odonata	Hemiphlebiidae	1	1
Od_Z_Isost	Odonata	Isostictidae	45	0.0222222222222222
Od_Z_Lesti	Odonata	Lestidae	150	0.00666666666666667
Od_Z_Megap	Odonata	Megapodagrionidae	285	0.00350877192982456
Od_Z_Peril	Odonata	Perilestidae	19	0.0526315789473684
Od_Z_Platycn	Odonata	Platycnemididae	222	0.0045045045045045
Od_Z_Platyst	Odonata	Platystictidae	189	0.00529100529100529
Od_Z_Polyt	Odonata	Polythoridae	58	0.0172413793103448
Od_Z_Proto	Odonata	Protoneuridae	240	0.00416666666666667
Od_Z_Pseuole	Odonata	Pseudolestidae	1	1
Od_Z_Pseuost	Odonata	Pseudostigmatidae	19	0.0526315789473684
Od_Z_Synle	Odonata	Synlestidae	33	0.0303030303030303
Or_C_Acrid	Orthoptera	Acrididae	6016	0.000166223404255319
Or_C_Choro	Orthoptera	Chorotypidae	160	0.00625
Or_C_Cylin	Orthoptera	Cylindrachetidae	16	0.0625
Or_C_Episa	Orthoptera	Episactidae	64	0.015625
Or_C_Eumas	Orthoptera	Eumastacidae	230	0.00434782608695652
Or_C_Eusch	Orthoptera	Euschmidtiidae	191	0.00523560209424084
Or_C_Lentu	Orthoptera	Lentulidae	35	0.0285714285714286
Or_C_Pamph	Orthoptera	Pamphagidae	448	0.00223214285714286
Or_C_Pneum	Orthoptera	Pneumoridae	17	0.0588235294117647
Or_C_Prosc	Orthoptera	Proscopiidae	214	0.00467289719626168
Or_C_Pyrgo	Orthoptera	Pyrgomorphidae	455	0.0021978021978022
Or_C_Rhipi	Orthoptera	Rhipipterygidae	69	0.0144927536231884
Or_C_Romal	Orthoptera	Romaleidae	465	0.0021505376344086
Or_C_Tanao	Orthoptera	Tanaoceridae	3	0.333333333333333
Or_C_Tetri	Orthoptera	Tetrigidae	1246	0.000802568218298555
Or_C_Theri	Orthoptera	Thericleidae	220	0.00454545454545455
Or_C_Trida	Orthoptera	Tridactylidae	132	0.00757575757575758
Or_C_Trigo	Orthoptera	Trigonopterygidae	16	0.0625
Or_C_Xyron	Orthoptera	Xyronotidae	4	0.25
Or_E_Anost	Orthoptera	Anostostomatidae	206	0.00485436893203883
Or_E_Gryllac	Orthoptera	Gryllacrididae	675	0.00148148148148148
Or_E_Grylli	Orthoptera	Gryllidae	4664	0.000214408233276158
Or_E_Gryllot	Orthoptera	Gryllotalpidae	100	0.01
Or_E_Myrme	Orthoptera	Myrmecophilidae	8	0.125
Or_E_Proph	Orthoptera	Prophalangopsidae	71	0.0140845070422535
Or_E_Rhaph	Orthoptera	Rhaphidophoridae	497	0.00201207243460765
Or_E_Steno	Orthoptera	Stenopelmatidae	28	0.0357142857142857
Or_E_Tetti	Orthoptera	Tettigoniidae	6827	0.000146477222791856
Pha_Agathe	Phasmatodea	Agathemeridae	8	0.125

Pha_Aschip	Phasmatodea	Aschiphasmatidae	96	0.0104166666666667
Pha_Bacill	Phasmatodea	Bacillidae	54	0.0185185185185185
Pha_Diaphe	Phasmatodea	Diapheromeridae	1210	0.000826446280991736
Pha_Heteronem	Phasmatodea	Heteronemiidae	79	0.0126582278481013
Pha_Heteropt	Phasmatodea	Heteropterygidae	103	0.00970873786407767
Pha_Phasma	Phasmatodea	Phasmatidae	991	0.00100908173562059
Pha_Phylli	Phasmatodea	Phylliidae	51	0.0196078431372549
Pha_Pseudo	Phasmatodea	Pseudophasmatidae	327	0.00305810397553517
Pha_Timema	Phasmatodea	Timematidae	21	0.0476190476190476
Pht_Boopid	Phthiraptera	Boopidae	55	0.0181818181818182
Pht_Echino	Phthiraptera	Echinophthiriidae	12	0.0833333333333333
Pht_Gyropi	Phthiraptera	Gyropidae	93	0.010752688172043
Pht_Haemat	Phthiraptera	Haematomyzidae	3	0.333333333333333
Pht_Heptap	Phthiraptera	Heptapsogasteridae	130	0.00769230769230769
Pht_Hoplop	Phthiraptera	Hoplopleuridae	157	0.00636942675159236
Pht_Laemob	Phthiraptera	Laemobothriidae	20	0.05
Pht_Linogn	Phthiraptera	Linognathidae	68	0.0147058823529412
Pht_Menopo	Phthiraptera	Menoponidae	1039	0.000962463907603465
Pht_Pedici	Phthiraptera	Pedicinidae	14	0.0714285714285714
Pht_Pedicu	Phthiraptera	Pediculidae	3	0.333333333333333
Pht_Philop	Phthiraptera	Philopteridae	2698	0.000370644922164566
Pht_Polypl	Phthiraptera	Polyplacidae	190	0.00526315789473684
Pht_Pthiri	Phthiraptera	Pthiridae	2	0.5
Pht_Ricini	Phthiraptera	Ricinidae	109	0.00917431192660551
Pht_Tricho	Phthiraptera	Trichodectidae	362	0.00276243093922652
Pl_Austrop	Plecoptera	Austroperlidae	15	0.0666666666666667
Pl_Capniid	Plecoptera	Capniidae	287	0.00348432055749129
Pl_Chlorop	Plecoptera	Chloroperlidae	187	0.0053475935828877
Pl_Diamphi	Plecoptera	Diamphipnoidae	6	0.166666666666667
Pl_Eusthen	Plecoptera	Eustheniidae	23	0.0434782608695652
Pl_Gripopt	Plecoptera	Gripopterygidae	270	0.0037037037037037
Pl_Leuctri	Plecoptera	Leuctridae	360	0.00277777777777778
Pl_Nemouri	Plecoptera	Nemouridae	674	0.00148367952522255
Pl_Notonem	Plecoptera	Notonemouridae	118	0.00847457627118644
Pl_Peltope	Plecoptera	Peltoperlidae	69	0.0144927536231884
Pl_Perlida	Plecoptera	Perlidae	965	0.00103626943005181
Pl_Perlodi	Plecoptera	Perlodidae	310	0.0032258064516129
Pl_Pterona	Plecoptera	Pteronarcyidae	12	0.0833333333333333
Pl_Scopuri	Plecoptera	Scopuridae	8	0.125
Pl_Stylope	Plecoptera	Styloperlidae	10	0.1
Pl_Taeniop	Plecoptera	Taeniopterygidae	103	0.00970873786407767
Pro_Aceren	Protura	Acerentomidae	118	0.00847457627118644
Pro_Berber	Protura	Berberentomidae	225	0.00444444444444444
Pro_Eosent	Protura	Eosentomidae	340	0.00294117647058824
Pro_Fujien	Protura	Fujientomidae	2	0.5
Pro_Hesper	Protura	Hesperentomidae	24	0.0416666666666667
Pro_Sinent	Protura	Sinentomidae	3	0.333333333333333
Ps_Amphien	Psocoptera	Amphientomidae	100	0.01
Ps_Amphips	Psocoptera	Amphipsocidae	180	0.00555555555555556

Ps_Archips	Psocoptera	Archipsocidae	81	0.0123456790123457
Ps_Asiopsc	Psocoptera	Asiopsocidae	14	0.0714285714285714
Ps_Caecili	Psocoptera	Caeciliusidae	566	0.00176678445229682
Ps_Calopso	Psocoptera	Calopsocidae	34	0.0294117647058824
Ps_Compsoc	Psocoptera	Compsocidae	2	0.5
Ps_Dasydem	Psocoptera	Dasydemellidae	30	0.0333333333333333
Ps_Ectopso	Psocoptera	Ectopsocidae	177	0.00564971751412429
Ps_Electre	Psocoptera	Electrentomidae	9	0.111111111111111
Ps_Elipsco	Psocoptera	Elipsocidae	129	0.00775193798449612
Ps_Epipsoc	Psocoptera	Epipsocidae	138	0.0072463768115942
Ps_Hemipso	Psocoptera	Hemipsocidae	24	0.0416666666666667
Ps_Lachesi	Psocoptera	Lachesillidae	271	0.003690036900369
Ps_Lepidop	Psocoptera	Lepidopsocidae	206	0.00485436893203883
Ps_Liposce	Psocoptera	Liposcelididae	181	0.00552486187845304
Ps_Mesopso	Psocoptera	Mesopsocidae	75	0.0133333333333333
Ps_Musapso	Psocoptera	Musapsocidae	9	0.111111111111111
Ps_Myopsoc	Psocoptera	Myopsocidae	159	0.00628930817610063
Ps_Pachytr	Psocoptera	Pachytroctidae	87	0.0114942528735632
Ps_Peripso	Psocoptera	Peripsocidae	235	0.00425531914893617
Ps_Philota	Psocoptera	Philotarsidae	111	0.00900900900900901
Ps_Prionog	Psocoptera	Prionoglarididae	7	0.142857142857143
Ps_Pseudoc	Psocoptera	Pseudocaeciliidae	300	0.00333333333333333
Ps_Psilops	Psocoptera	Psilopsocidae	7	0.142857142857143
Ps_Pscocid	Psocoptera	Psocidae	899	0.00111234705228031
Ps_Psoquil	Psocoptera	Psoquillidae	27	0.037037037037037
Ps_Psyllip	Psocoptera	Psyllipsocidae	26	0.0384615384615385
Ps_Sphaero	Psocoptera	Sphaeropsocidae	15	0.0666666666666667
Ps_Stenops	Psocoptera	Stenopsocidae	95	0.0105263157894737
Psc_Tricho	Psocoptera	Trichopsocidae	11	0.0909090909090909
Ps_Troctop	Psocoptera	Troctopsocidae	22	0.0454545454545455
Ps_Trogiid	Psocoptera	Trogiidae	52	0.0192307692307692
Rap_Inocel	Raphidioptera	Inocelliidae	25	0.04
Rap_Raphid	Raphidioptera	Raphidiidae	200	0.005
Si_Ceratop	Siphonaptera	Ceratophyllidae	403	0.00248138957816377
Si_Chimero	Siphonaptera	Chimaeropsyllidae	23	0.0434782608695652
Si_Coptops	Siphonaptera	Coptopsyllidae	19	0.0526315789473684
Si_Ctenoph	Siphonaptera	Ctenophthalmidae	664	0.00150602409638554
Si_Hystri	Siphonaptera	Hystrichopsyllidae	35	0.0285714285714286
Si_Ischnop	Siphonaptera	Ischnopsyllidae	125	0.008
Si_Leptops	Siphonaptera	Leptopsyllidae	260	0.00384615384615385
Si_Lycopsy	Siphonaptera	Lycopsyllidae	8	0.125
Si_Macrops	Siphonaptera	Macropsyllidae	2	0.5
Si_Pulicid	Siphonaptera	Pulicidae	167	0.00598802395209581
Si_Pygiops	Siphonaptera	Pygiopsyllidae	48	0.0208333333333333
Si_Rhopalo	Siphonaptera	Rhopalopsyllidae	126	0.00793650793650794
Si_Stephan	Siphonaptera	Stephanocircidae	51	0.0196078431372549
Si_Stivali	Siphonaptera	Stivaliidae	111	0.00900900900900901
Si_Vermips	Siphonaptera	Vermipsyllidae	36	0.0277777777777778
Strepsipte	Strepsiptera	Strepsiptera	590	0.00169491525423729

Thy_Aeolot	Thysanoptera	Aeolothripidae	201	0.00497512437810945
Thy_Hetero	Thysanoptera	Heterothripidae	76	0.0131578947368421
Thy_Phlaeo	Thysanoptera	Phlaeothripidae	3532	0.00028312570781427
Thy_Thripi	Thysanoptera	Thripidae	2066	0.000484027105517909
Tr_Anomalo	Trichoptera	Anomalopsychidae	27	0.037037037037037
Tr_Apatani	Trichoptera	Apataniidae	203	0.00492610837438424
Tr_Atriple	Trichoptera	Atriplectididae	6	0.166666666666667
Tr_Beraeid	Trichoptera	Beraeidae	57	0.0175438596491228
Tr_Brachyc	Trichoptera	Brachycentridae	111	0.00900900900900901
Tr_Calamoc	Trichoptera	Calamoceratidae	182	0.00549450549450549
Tr_Calocid	Trichoptera	Calocidae	23	0.0434782608695652
Tr_Chatham	Trichoptera	Chathamiidae	5	0.2
Tr_Conoesu	Trichoptera	Conoesucidae	43	0.0232558139534884
Tr_Dipseud	Trichoptera	Dipseudopsidae	114	0.0087719298245614
Tr_Ecnomid	Trichoptera	Ecnomidae	469	0.00213219616204691
Tr_Glossos	Trichoptera	Glossosomatidae	682	0.00146627565982405
Tr_Goerida	Trichoptera	Goeridae	184	0.00543478260869565
Tr_Helicophi	Trichoptera	Helicophidae	44	0.0227272727272727
Tr_Helicopsy	Trichoptera	Helicopsychidae	269	0.00371747211895911
Tr_Hydrobi	Trichoptera	Hydrobiosidae	407	0.00245700245700246
Tr_Hydrops	Trichoptera	Hydropsychidae	1808	0.000553097345132743
Tr_Hydropt	Trichoptera	Hydroptilidae	2124	0.000470809792843691
Tr_Hydrosa	Trichoptera	Hydrosalpingidae	1	1
Tr_Kokirii	Trichoptera	Kokiriidae	15	0.0666666666666667
Tr_Lepidos	Trichoptera	Lepidostomatidae	471	0.00212314225053079
Tr_Leptoce	Trichoptera	Leptoceridae	2020	0.000495049504950495
Tr_Limneph	Trichoptera	Limnephilidae	880	0.00113636363636364
Tr_Limnoce	Trichoptera	Limnocentropodidae	15	0.0666666666666667
Tr_Molanni	Trichoptera	Molannidae	41	0.024390243902439
Tr_Odontoc	Trichoptera	Odontoceridae	154	0.00649350649350649
Tr_Oecones	Trichoptera	Oeconesidae	18	0.0555555555555556
Tr_Philopo	Trichoptera	Philopotamidae	1168	0.000856164383561644
Tr_Philorh	Trichoptera	Philorheithridae	30	0.0333333333333333
Tr_Phrygane	Trichoptera	Phryganeidae	84	0.0119047619047619
Tr_Phryganop	Trichoptera	Phryganopsychidae	4	0.25
Tr_Pisulii	Trichoptera	Pisuliidae	19	0.0526315789473684
Tr_Polycen	Trichoptera	Polycentropodidae	806	0.00124069478908189
Tr_Psychom	Trichoptera	Psychomyiidae	522	0.00191570881226054
Tr_Rhyacop	Trichoptera	Rhyacophilidae	774	0.00129198966408269
Tr_Sericos	Trichoptera	Sericostomatidae	107	0.00934579439252336
Tr_Stenops	Trichoptera	Stenopsychidae	94	0.0106382978723404
Tr_Tasimii	Trichoptera	Tasimiidae	9	0.111111111111111
Tr_Uenoida	Trichoptera	Uenoidae	31	0.032258064516129
Tr_Xiphoce	Trichoptera	Xiphocentronidae	172	0.00581395348837209
Zoraptera	Zoraptera	Zoraptera	35	0.0285714285714286
Zy_Lepidot	Zygentoma	Lepidotrichidae	1	1
Zy_Lepisma	Zygentoma	Lepismatidae	200	0.005
Zy_Nicolet	Zygentoma	Nicoletiidae	30	0.0333333333333333

Figure S1. Global pattern of insect diversification based on the fossil record for the orders (a) and the families (b). Origination (blue) and extinction (red) rates were estimated using PyRate constrained with time bins as defined by 10-Myr bins. Solid lines indicate mean posterior rates, whereas the shaded areas show 95% HPD intervals. Net diversification rates (black) are defined as origination minus extinction. The vertical lines indicate the boundaries between geological boundaries and major mass extinction events. Abbreviations as in Fig. 2.

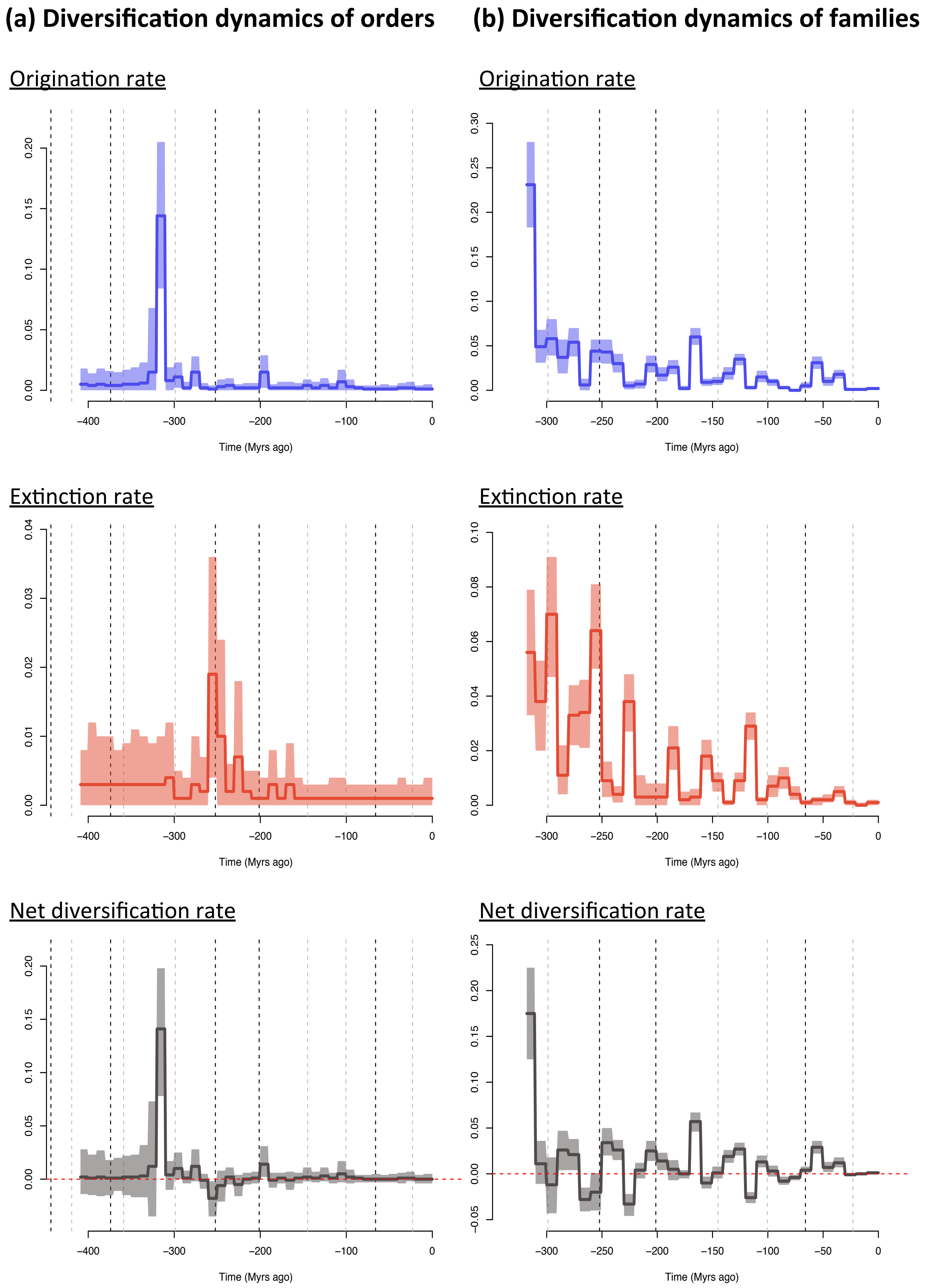

Figure S2. Convergence of the BAMM analysis with the chronogram. a) The stationary of the MCMC before applying a burn-in. b) The posterior distribution of number of shifts estimated before applying a burn-in.

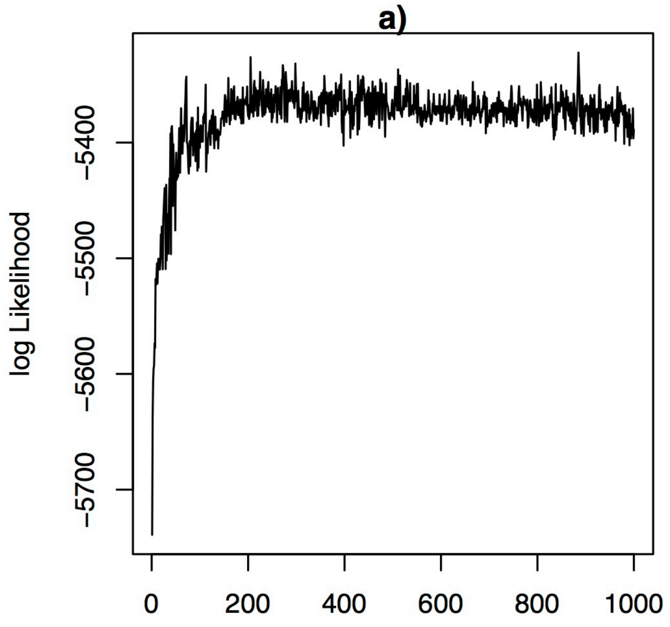

Figure S3. Frequency distribution of distinct macroevolutionary rate regimes estimated using BAMM. a) Prior distribution of the number of distinct processes. b) Posterior distribution of the number of distinct processes (including the root process). A model with eight shifts outperforms the other models.

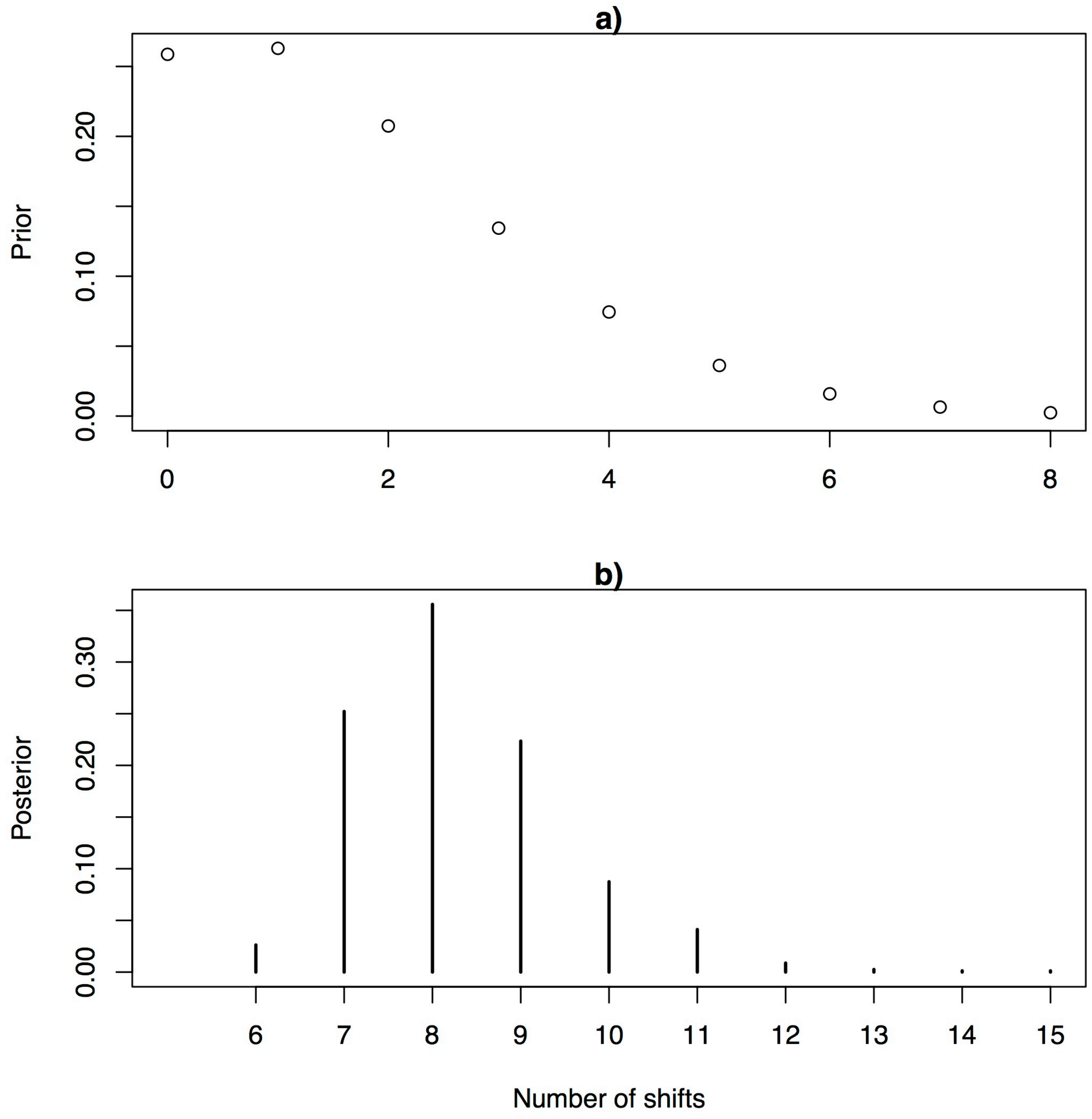

Figure S4. Credible set of configuration shifts of insects inferred with BAMM.

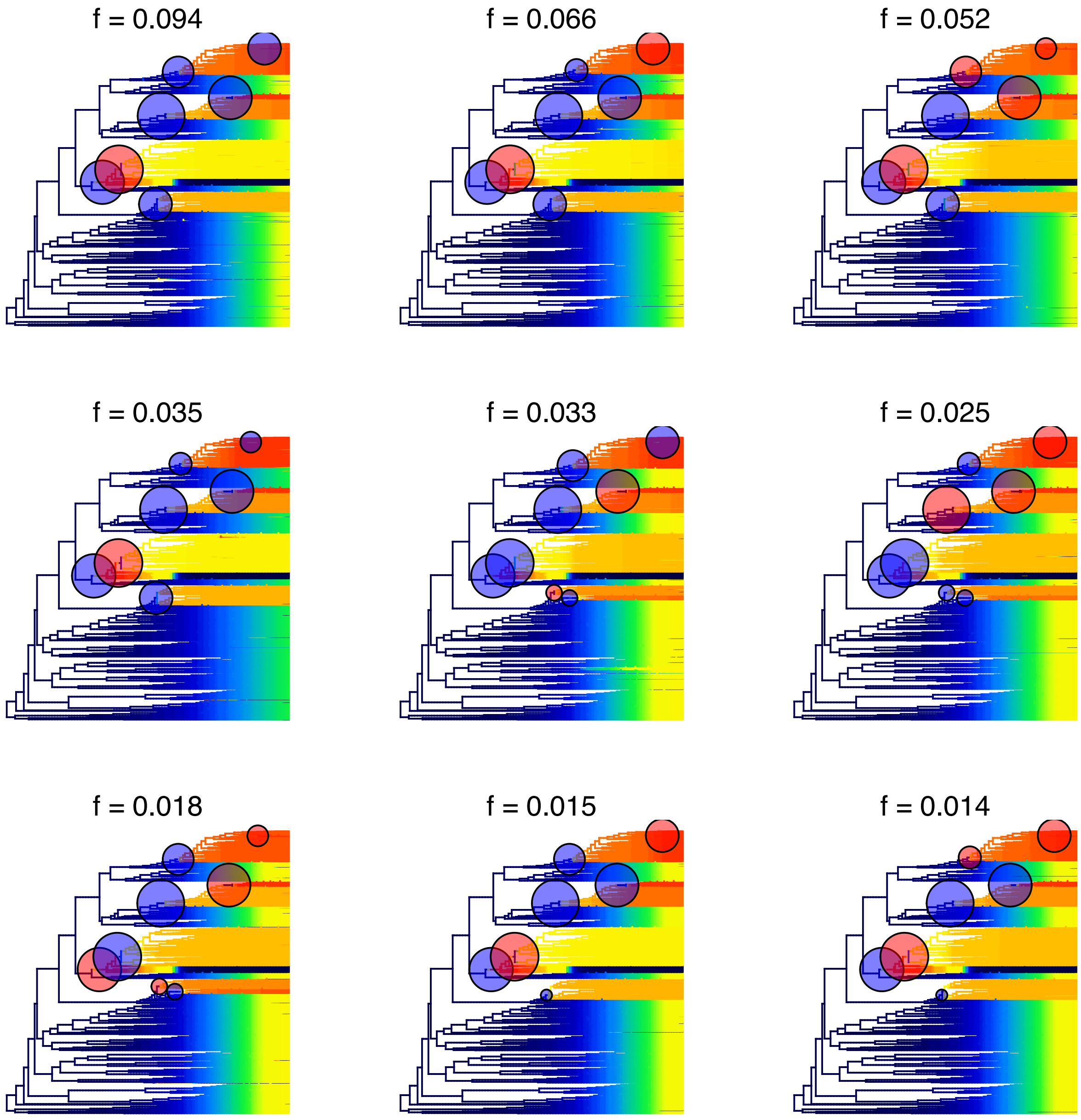

Figure S5. The best shift configuration inferred with BAMM for insects.

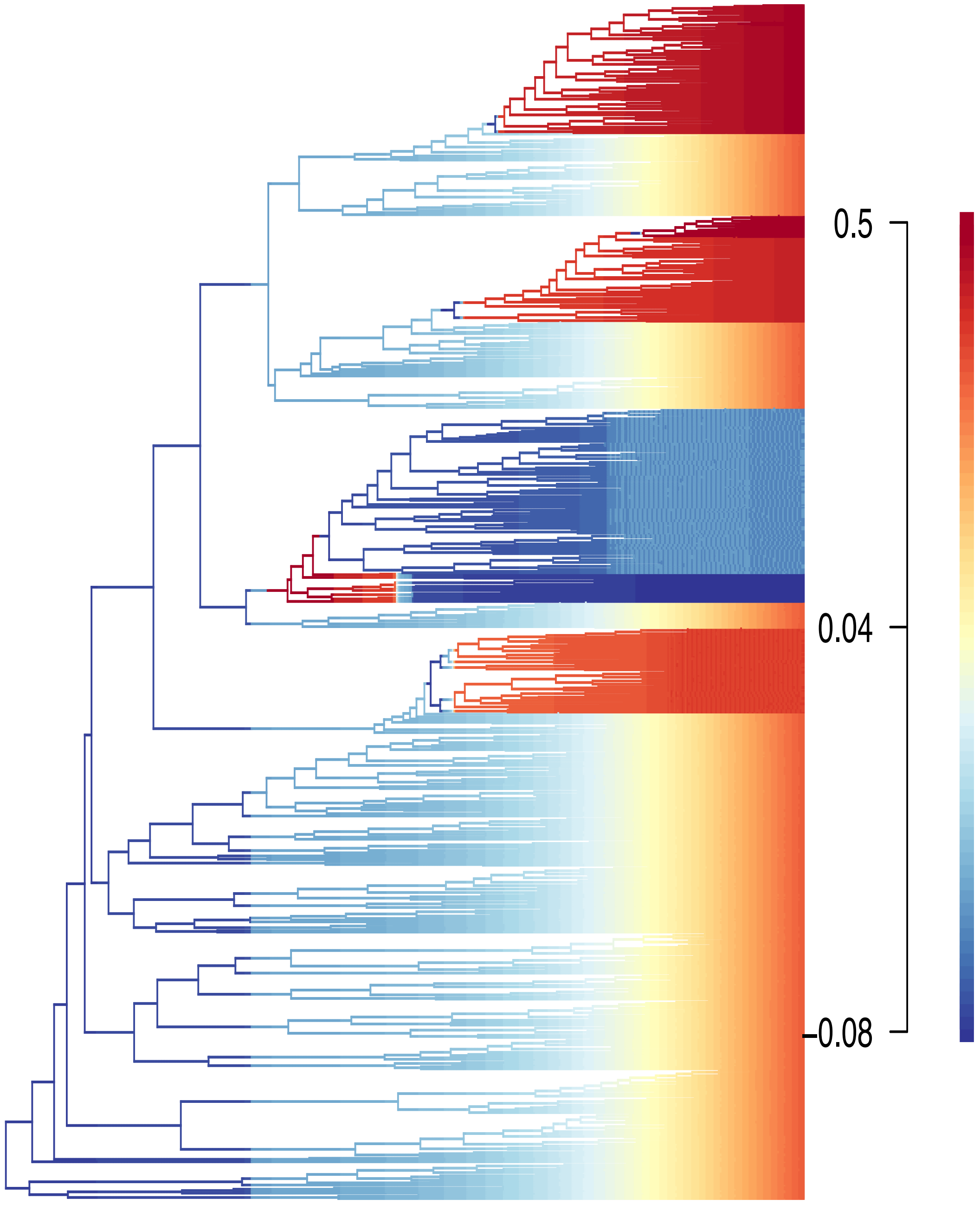

Figure S6. Global pattern of diversification rates over the insect evolution (top, speciation rate; middle, extinction rate; bottom, net diversification rate).

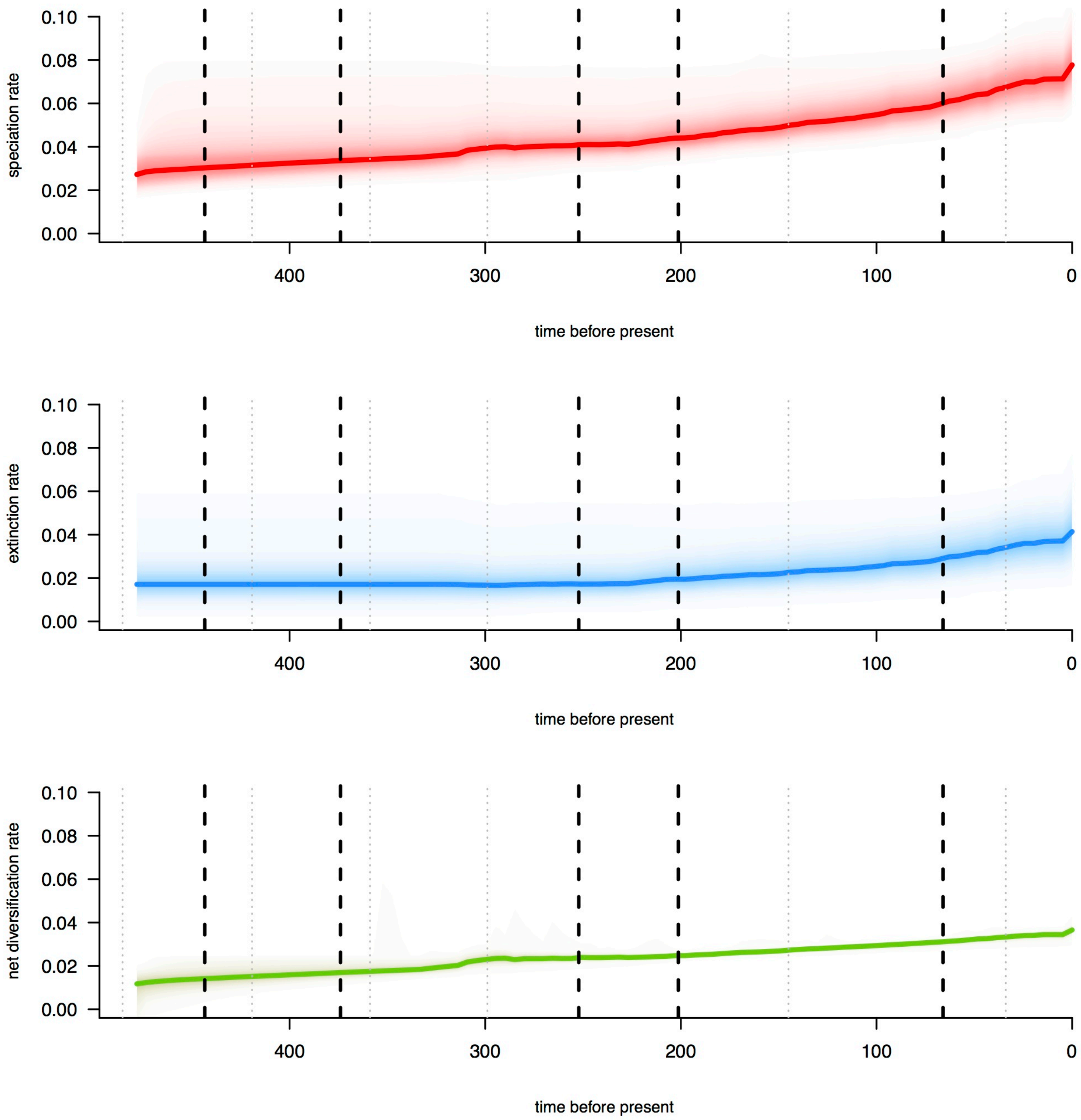

Figure S7. Pattern of diversification rates for the five richest insect orders and the rest of insect (curves represent the net diversification rates).

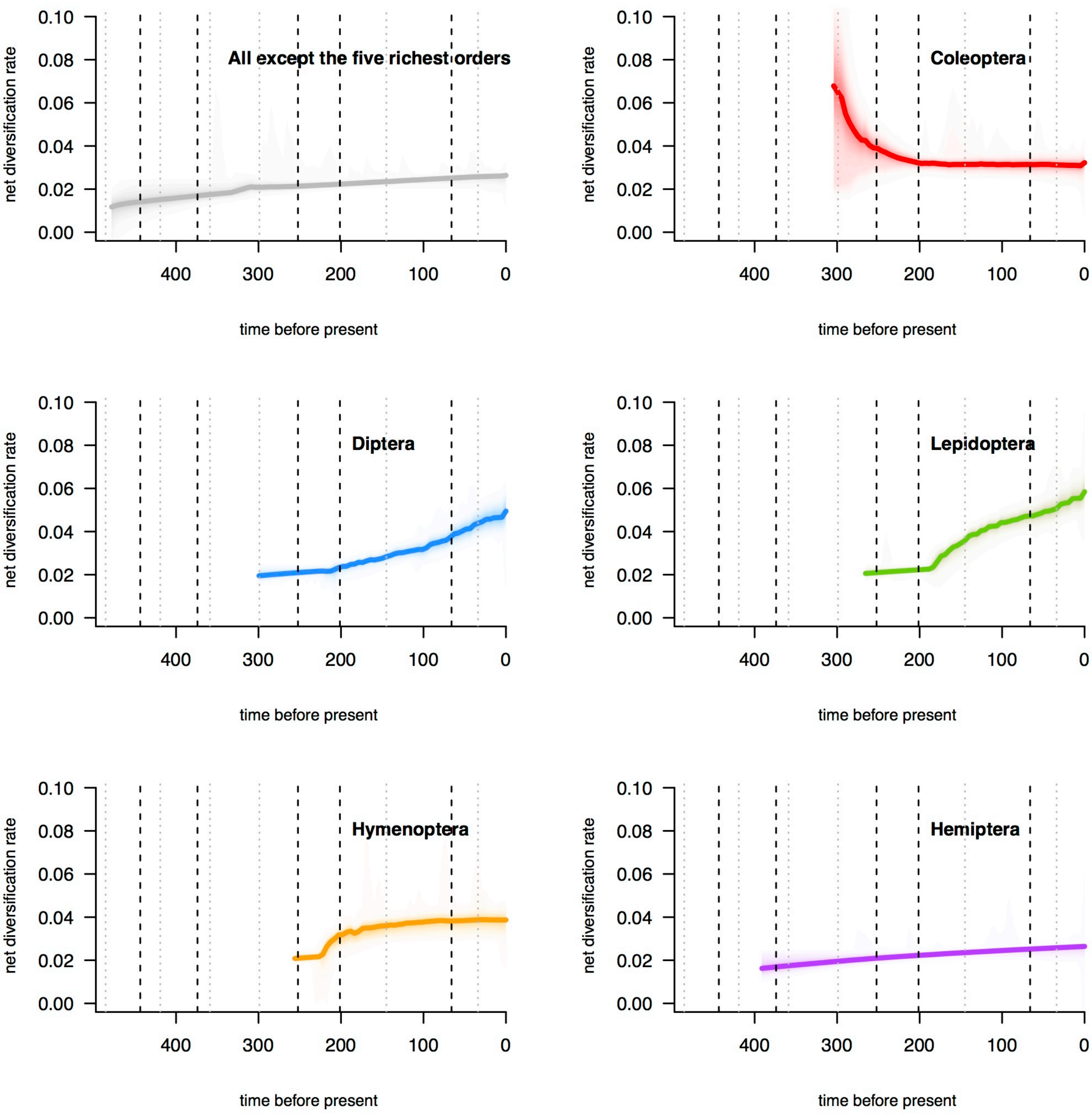

Figure S8. Contrasting the diversification rates of non-Pterygota versus Pterygota (top plots), and the diversification rates of non-Holometabola versus Holometabola (bottom plots). One can see that no significant increase of diversification is detected at the origin of Pterygota, nor at the origin of Holometabola.

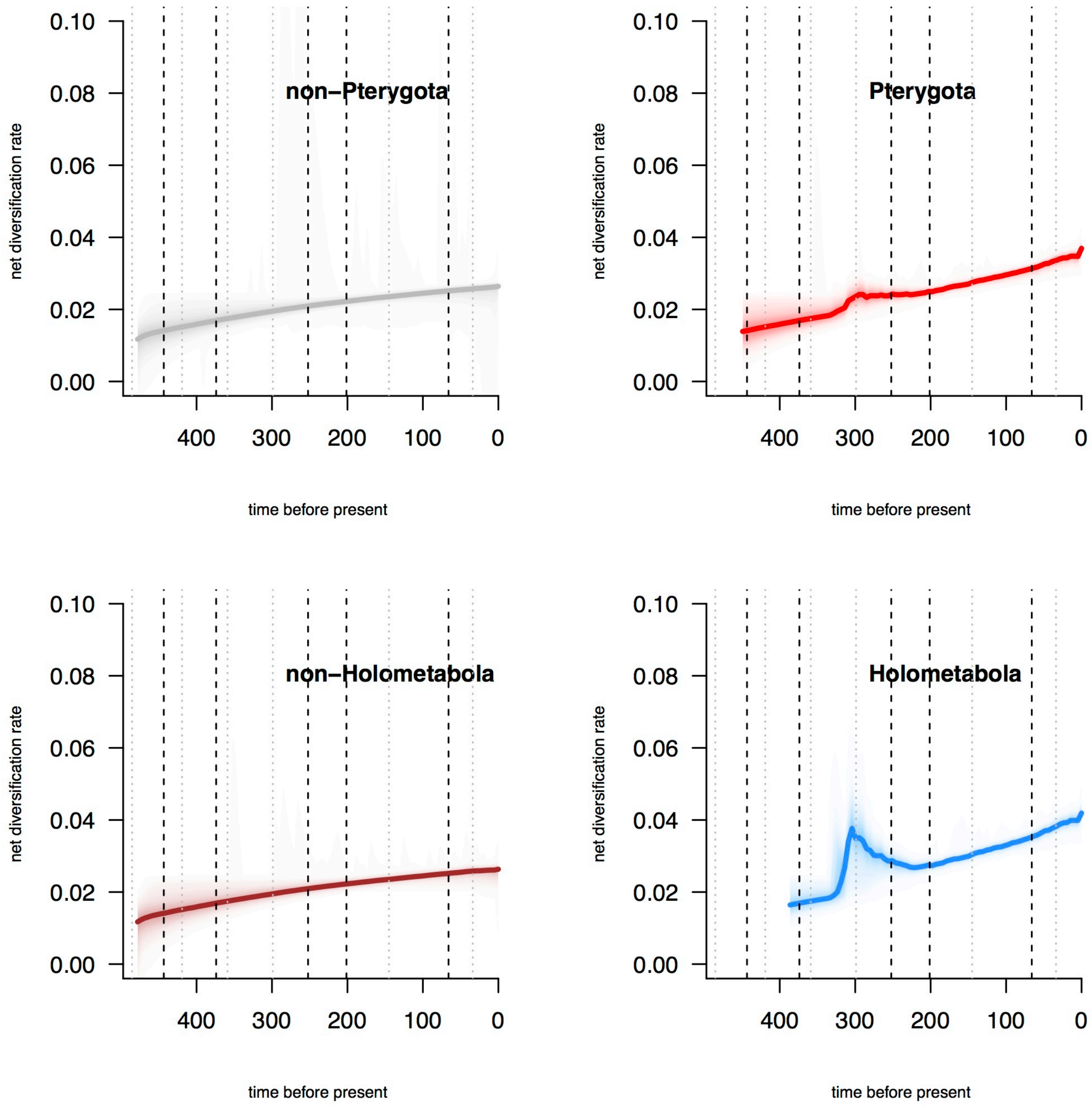

Supplement: Supplementary Information [file srep19208-s4.pdf]
